# A comparative study of small RNAs in *Toxoplasma gondii* of distinct genotypes

**DOI:** 10.1186/1756-3305-5-186

**Published:** 2012-09-03

**Authors:** Jielin Wang, Xiaolei Liu, Boyin Jia, Huijun Lu, Shuai Peng, Xianyu Piao, Nan Hou, Pengfei Cai, Jigang Yin, Ning Jiang, Qijun Chen

**Affiliations:** 1 Key Laboratory of Zoonosis, Ministry of Education, Jilin University, Xi An Da Lu 5333, Changchun, 130062, China; 2 MOH Key Laboratory of Systems Biology of Pathogens, Institute of Pathogen Biology, Chinese Academy of Medical Sciences & Peking Union Medical College, Beijing, China

## Abstract

**Background:**

*Toxoplasma gondii* is an intracellular parasite with a significant impact on human health. Inside the mammalian and avian hosts, the parasite can undergo rapid development or remain inactive in the cysts. The mechanism that regulates parasite proliferation has not been fully understood. Small noncoding RNAs (sncRNA) such as microRNAs (miRNAs) are endogenous regulatory factors that can modulate cell differentiation and development. It is anticipated that hundreds of miRNAs regulate the expression of thousands of genes in a single organism. SncRNAs have been identified in *T. gondii*, however the profiles of sncRNAs expression and their potential regulatory function in parasites of distinct genotypes has largely been unknown.

**Methods:**

The transcription profiles of miRNAs in the two genetically distinct strains, RH and ME49, of *T. gondii* were investigated and compared by a high-through-put RNA sequencing technique and systematic bioinformatics analysis. The expression of some of the miRNAs was confirmed by Northern blot analysis.

**Results:**

1,083,320 unique sequences were obtained. Of which, 17 conserved miRNAs related to 2 metazoan miRNA families and 339 novel miRNAs were identified. A total of 175 miRNAs showed strain-specific expression, of which 155 miRNAs were up-regulated in RH strain and 20 miRNAs were up-regulated in ME49 strain. Strain-specific expression of miRNAs in *T. gondii* could be due to activation of specific genes at different genomic loci or due to arm-switching of the same pre-miRNA duplex.

**Conclusions:**

Evidence for the differential expression of miRNAs in the two genetically distinct strains of *T. gondii* has been identified and defined. MiRNAs of *T. gondii* are more species-specific as compared to other organisms, which can be developed as diagnostic biomarkers for toxoplasmosis. The data also provide a framework for future studies on RNAi-dependent regulatory mechanisms in the zoonotic parasite.

## Background

*Toxoplasma gondii* is an obligatory intracellular parasite that infects a wide range of hosts,including humans, animals and birds. It is considered to be one of the most widely distributed protozoan parasite with a sero-prevalence in humans of up to 30% worldwide [1]. *T. gondii* is the etiological agent of toxoplasmosis which can be either life-threatening or long-term chronic infection.

The life cycle of *Toxoplasma gondii* is unusual in that the parasite is capable of indefinite proliferation in the hosts with either a sexual or an asexual cycle. The sexual cycle occurs only in the hosts of a feline species. The asexual cycle can occur in virtually any warm-blooded animals, which act as the intermediate hosts, ranging from chicken to baleen whales and humans. In the parasite’s life cycle, there are three fundamental morphotypes, named tachyzoites, bradyzoites and sporozoites. The development of *T. gondii* in the intermediate host involves an initial phase with a rapid proliferation of the tachyzoites, followed by the formation of tissue cysts containing slowly dividing or resting bradyzoites.

While the global population structure of *T. gondii* awaits further elucidation [2], three clonal lineages (Type I, II, and III) of *T. gondii* which comprise the majority of strains in both North America and Europe [3-5]. Recently, a fourth clonal lineage, designated haplogroup 12, has been identified based on isolates that are common in wild animals in the United States [6]. The virulence of *T. gondii* is normally defined based on the LD50 in mice. Type I strain has been regarded as a more virulent strain in mice with an LD50 <10 [4,7]. In contrast, type II (e.g., ME49) and type III strains are less lethal in mice (LD50 > 100) and the infections are usually less severe or asymptomatic [8]. The clonal lineages also differ in a number of phenotypes such as growth rate, efficiency in migration and transmigration in tissues [7,9].

Several studies have been carried out with aims to determine differential gene expression at various lifecycle stages of *T. gondii*[10-13]. Likewise, efforts were taken to reveal the factors that may modulate the virulence of *T. gondii*. ROP5, ROP16 and ROP18, which are rhoptry-derived factors, and their expression was found to correlate with parasite virulence [14-18]. Identification of strain-specific regulatory elements responsible for the distinct genotype will not only facilitate our understanding of parasite biology, but may also elucidate the association between genetically defined subpopulations (strains) and disease severity.

In recent years, the discovery of numerous small RNAs has increased our knowledge in post-transcriptional gene regulation in cell development and other biological processes. Small RNAs, such as microRNA (miRNA), small interfering RNA (siRNA), and Piwi-associated RNA (piRNA), are regulatory elements that can modulate gene expression at the post-transcriptional level. MiRNAs, a class of ≈ 22 nucleotide small-RNA sequences that participate in the post-transcriptional regulation of gene expression [19], have been known to play critical roles in diverse biological processes, including development, viral defense, metabolism, and apoptosis [20-23]. The primary transcripts (pri-miRNAs) of miRNA genes are generated by either RNA polymerase II [24] or RNA polymerase III [25]. A single pri-miRNA may contain from one to six miRNA precursors. They are processed by RNase complexes (Drosha and DGCR8) into ≈ 70 nucleotide fragments with a stem-loop structure called pre-miRNA. The pre-miRNAs are then exported to the cytoplasm by Exportin-5 [26] and the hairpin is cleaved by the RNase III enzyme (Dicer) to miRNA duplexes [27]. A single miRNA can silence a number of genes while one gene can be targeted by several miRNAs [28,29]. Previous studies have found that *T. gondii* has a complete machinery for small RNA generation and small RNA-mediated gene regulation [30]. There are mainly two classes of small regulatory RNAs derived from the *T. gondii* genome, namely miRNA and rdsRNA. And it was found that rdsRNAs were consistently more abundant in the highly virulent *Toxoplasma* isotype-I (RH) than in other two strains (RPU and CTG). Further, it was recently reported that the Argonaute of *T. gondii* (TgAgo) is methylated and its activity is Mg^2+^-dependent [31]. However the differential expression of miRNA in parasites of distinct genotypes has not been well investigated.

In this study, we systematically studied the expression of miRNAs in type I (RH) and type II strain (ME49) *T. gondii* by high-through-put RNA sequencing technology and bioinformatic analysis. A number of *T. gondii* specific miRNAs was identified. Meanwhile, differentially expressed miRNAs between the two strains of *T. gondii* were detected. The results demonstrated profound differences between the two strains of *T. gondii* in miRNA expression.

## Methods

### Parasites

Tachyzoites of *T. gondii* RH strain and ME49 strain were propagated in mice. The tachyzoites were purified by density-gradient centrifugation on Percoll [32]. The study of using laboratory animals was reviewed and approved by the Ethical Committee of Jilin University.

### RNA isolation

Total RNA of *T. gondii* (both RH and ME49 strain) was prepared using Trizol reagent (Invitrogen, SF, USA) according to the manufacturer’s protocol. The integrity of total RNA was examined by standard agarose gel electrophoresis, and RNA purity was reflected by the 260/280 nm absorbance ratio and the concentration was determined using a Nanodrop 1000 machine (Thermo Scientific CA, USA). The purified total RNA was stored at-80°C until use.

### Construction of small RNA libraries and sequencing

For small RNA library construction and deep sequencing, RNA samples from RH and ME49 strains of *T. gondii* were prepared as follows: for each strain, equal quantities (10 μg) of RNA isolated from tachyzoites were pooled. Small RNA molecules in the range of 18–30 nt RNA was purified after polyacrylamide gel electrophoresis (PAGE) and ligated with proprietary adapters to the 5^′^ and 3^′^ termini. The samples were used as templates for cDNA synthesis. The cDNA was amplified to produce sequencing libraries which were subjected to Solexa’s sequencing-by-synthesis method. Sequencing was carried out at the Beijing Genomics Institute (BGI, China). Two separated runs with two batches of RNAs were carried out in the sequencing. Only sequences with high quality were included in the analysis.

### Mapping sequence reads to the reference genome

Individual sequence reads with the base quality scores were produced by Solexa. Clean reads were obtained after removing of the low quality reads, such as adaptor null reads, insert null reads, 5^′^ adaptor contaminants, and reads with polyA tail and ambiguous bases. Adapter sequences were trimmed from both ends of clean reads before analysis. All identical sequences were counted and eliminated from the initial data set. The resulting set of unique sequences with associated read counts was referred to as sequence tags. The unique reads were mapped onto the *T. gondii* genome (http://www.toxodb.org) using the program SOAP (http://soap.genomics.org.cn) [33].

### Bioinformatic analysis of *T. gondii* small RNAs

Sequences were, at the first step, searched against Metazoa mature miRNA of Sanger miRBase allowing two mismatches to identify homologs of known Metazoa miRNAs using the program Patscan [34]. Sequence tags of more than 5 reads that matched perfectly or near-perfectly (no more than 2 mismatches and mismatch not positioned in seed region) to metazoan mature miRNAs were assumed to be conserved miRNA candidates. Secondly, the remaining sequences were screened against the non-coding RNA database [35] and the *T. gondii* genome database, and some non-coding RNAs such as rRNA, tRNA and snoRNAs were identified and filtered out for further analysis. Thirdly, We searched for the inverted repeats (step loops or hairpin structure) among the remained sequences which did not match to the miRNA database by using the software Einverted of Emboss [36] with the parameters following parameters: threshold = 30, match score = 3, mismatch score = 3, gap penalty = 6, and maximum repeat length = 240 as described [37]. Each inverted repeat was extended 10 nt on each side, and the secondary structure of the inverted repeat was predicted by RNAfold [38]. Unique reads in the inverted repeats were evaluated by MirCheck [37] using modified parameters that are more suitable for organisms such as worms and protozoa. Finally, miRNA precursors that passed MirCheck were inspected manually in order to remove the false prediction (Additional file 1: Figure S1). Further, miRNAs with the same sequences derived from pre-miRNAs (the sequences may not be completely the same) located at different genomic loci are indicated with an additional dash-number suffix. Sequences of *T. gondii*-specific miRNAs have been deposited in the miRNA database (miRBase).

MiRNAs with statistically significant differences in relative abundance (as reflected by TPM values) between the two libraries (corresponding to the two distinct strains of the parasites) were analyzed with IDEG6 and edger [39]. We used the general Chi-square method as it is most frequently used in such analysis [40,41]. MiRNA with a P value ≤ 0.01 were deemed to be significantly different between the samples of the two distinct strains of parasites.

### Northern blot analysis of miRNA expression

Twenty micrograms of total RNA was loaded in each well of a 12.5% polyacrylamide gel containing 42% urea and run in 0.5× TBE buffer. Following the electrophoresis, RNA was transferred by capillary transferring to a Hybond-N + nylon membranes (GE Healthsystems, Uppsala, Sweden). After UV cross-linking, the membranes were baked for 1 h at 80°C. Probes complementary to small RNA sequences were end-labeled with DIG at 5^′^ Termini (TaKaRa, Dalian, China). Pre-hybridization of the membrane was performed by overnight incubation at 53°C followed by hybridization overnight at the same temperature in Northernmax Hybridization buffer (Ambion, CA, USA). After hybridization, the membranes were washed four times for 30 min in 2×SSC, 0.05% SDS and twice for 15 min in 0.1×SSC, 0.1% SDS at RT. Chemiluminescent signal was detected using a DIG Detection Kit (Roche, Germany) following the manufacturer’s instructions. The oligonucleotide probes used for hybridization are as follows:

Tgo-novel-40 probe: TTCCTG*ATCCTA*TTTAG*CAG*GT

Tgo-novel-12-1 probe: CCA*CTTCAG*TCTTCA*AAG*TTCT

Tgo-novel-41 probe: AGGG*CGG*ACTG*GCTG*CTTCCAGCA

Tgo-novel-1-1-3p probe: GACAG*TGCTCG*GGTCCG*CAACA*CGCC

Tgo-novel-14-2 probe: ACA*CTT*CCCTT*CTCG*CCG

(LNA (locked nucleic acid) substitutions are indicated by a “*”).

## Results

### An overview of the small RNA sequencing results

**Figure 1:**
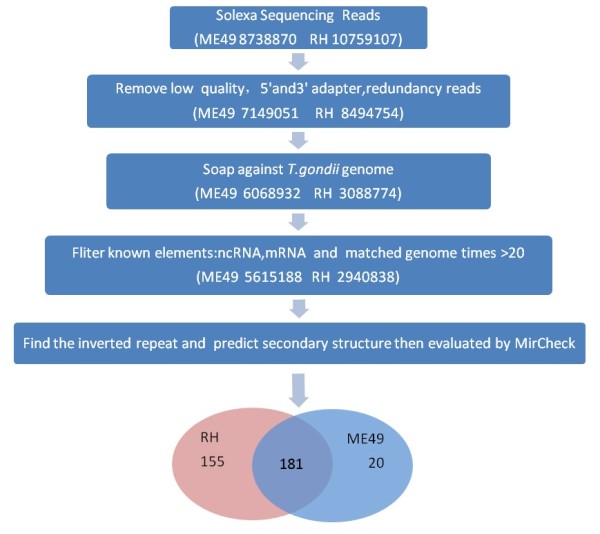
**Work flow for profiling of small RNAs after high-through-put sequencing.** Approaches and numbers of sequence tags obtained after each step were shown for both strains. The simple pie chart shows the number of strain-specific and common miRNAs identified in the two libraries. More miRNAs (155) were identified in RH strain than that in ME49. The number of miRNAs that commonly expressed in the two strains is 181.

A total of 8,738,870 and 10,759,107 sequence reads were obtained from the ME49 and RH libraries, respectively. There is no major difference in total numbers of low and high quality reads between the two libraries (Additional file 2: Table S1). After removal of the low quality reads, 7,149,051 (ME49) and 8,494,754 (RH) clean reads were obtained, which contained 247,346 (ME49) and 867,853 (RH) library-specific (unique) reads, respectively (Figure 1).

**Figure 2:**
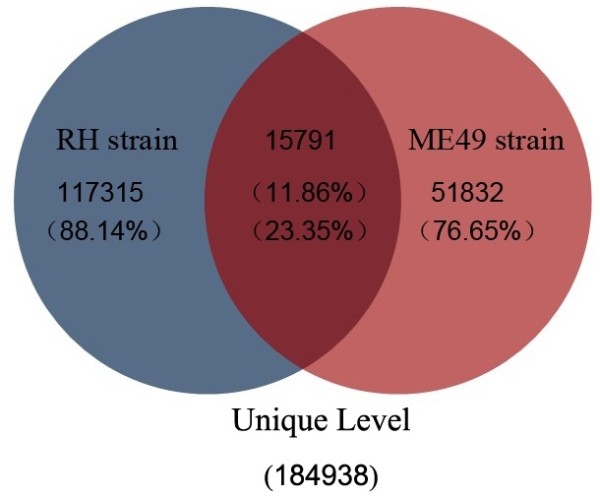
**Comparison of small RNAs sequences identified in the two strains of* T. gondii *at unique level.** Of the small RNAs identified in the two strains of T. gondii, strain-specific small RNAs were much more (88.14% of RH strain and 76.65% of ME49 strain) than that shared by the two strains (11.86% of RH strain and 23.35% of ME49 strain).

**Figure 3:**
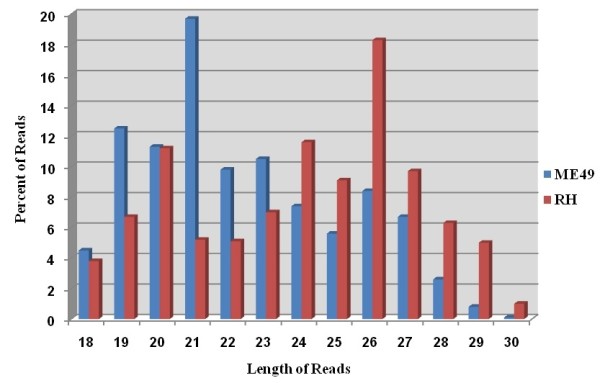
**The length distribution of small ncRNAs in the library of ME49 (blue) and RH (red) of* T. gondii *.** The dominant sncRNA expressed in ME49 strain is 21 nt in length, while the 26 nt sncRNAs were dominant in RH strain.

**Figure 4:**
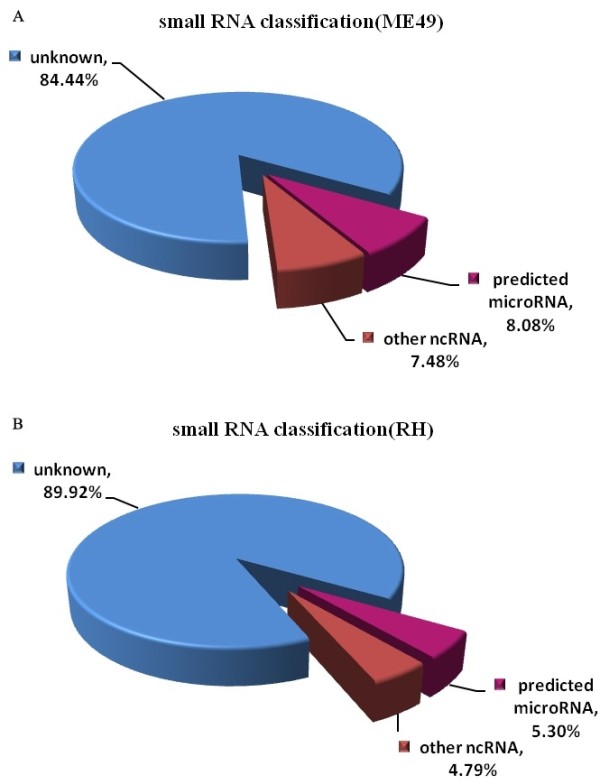
**Percentages of small ncRNAs in the two libraries (A. ME 49 strain, B. RH strain) of* T. gondii. *** A majority of the sncRNAs in the two libraries is unknown. The proportion of miRNAs in ME49 and RH strain is 8.08% and 5.30% respectively.

The clean unique reads described above were mapped to the draft genome of *T. gondii* (http://toxodb.org/toxo/showApplication.do) [33]. 6,068,932 and 3,088,774 non-redundant total reads from ME49 and RH were perfectly mapped onto the *T. gondii* genome, respectively (Additional file 3: Table S2). Among these reads, 51,832 (76.65% of total reads in ME49 library) displayed strain-specificity in ME49, while in RH the number of strain-specific reads was 117,315 (88.14% of total reads in RH library), the number of small RNA reads identified in the two strains was 15,791, which accounted for 11.86% and 23.35% of the total reads of RH and ME49, respectively (Figure 2). The length of small RNAs varied from 18 to 30 nt in the two strains. However, the length distributions of small RNAs were significantly different (Figure 3, Additional 4: Figure S2 and Additional file 5: Table S3). A majority of small RNAs in ME49 was 21 nt in length, whereas small RNAs of 26 nt were the most abundant in RH strain. The small RNAs were further categorized into, based on the sequence characteristics, rRNAs, tRNAs, small nuclear RNAs (snRNAs) and other ncRNA after BLASTN searches against the Sanger Rfam database release 9.0 (Additional file 3: Table S2). The proportions of small ncRNAs in the two libraries (ME49 and RH) were shown in Figure 2 and Figure 4A, B.

### Identification of miRNAs in *T. gondii*

**Figure 5:**
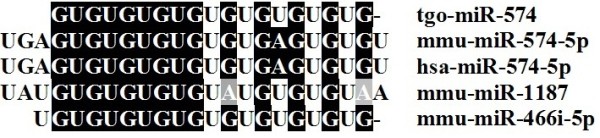
**Alignment of tgo-miR-574 sequence with homologues from other organisms.** The seed sequences are shadowed in dark colour. Mmu-miR-574-5p, mmu-miR-1187 and mmu-Mir-466i-5p are miRNAs identified in mice. Has-miR-574-5p is a miRNA identified in humans.

The proportions of miRNAs in the two libraries accounted between 5-8% of the total reads of the small ncRNAs (Figure 4 A, B). In total, 17 conserved miRNAs were found based on the consensus ‘seed’ region (2–8 nt in 5^′^ end of mature miRNA) identical to that of Homo sapiens, Mus musculus and Pongo pygmaeus (Figure 5), which were recognized by mRNAs through base pairing comparison [42]. In addition to the conserved miRNAs, we also found 339 species-specific miRNAs (novel miRNAs) of *T. gondii*. Moreover, 20 (5.6%) of 356 miRNAs were from the ME49 library, while 155 (43.6%) were from RH library, and 181 (50.8%) of the miRNAs were found in both libraries (Figure 1, Additional file 6: Table S4 and Additional 7: Table S5). The number of strain-specific miRNAs in RH strain was more than that in ME49 strain.

### Characterization of the expression of *T. gondii* miRNAs

Based on their genomic locations, *T. gondii* miRNAs were categorized into three types (Additional file 6: Table S4 and Additional file 7: Table S5) named intronic, intergenic and UTR-derived miRNAs. The numbers of miRNAs derived from the 3 genomic regions were 33, 305 and 18 respectively. No miRNA genes were found to be located in exons. Thus miRNA genes were predominantly intergenic in *T. gondii*. This observation was in agreement with a previous study on Schistosoma japonicum which suggested that most miRNA genes have their own control elements (or promoters) in the genome [43]. Further, it was found that a miRNA can be generated from several pre-miRNAs encoded by genes located in different genomic loci. Thus miRNAs with the same sequence but derived from different pre-miRNAs were named with an additional dash-number suffix (Additional file 6: Table S4 and Additional file 7: Table S5).

Comparison of the novel miRNAs expressed in ME49 and RH revealed strain-specific expression patterns in the two distinct strains of *T. gondii*. For instance, the number of reads of tgo-novel-40 in ME49 was 4000 times more than that in RH. Similarly, the expression level of tgo-novel-1-1-5p, tgo-novel-12-1, tgo-novel-41 and tgo-novel-15-1 was significantly higher in ME49 strain. On the contrary, tgo-novel-14-2, was highly expressed in RH, the reads number was about 10 times as much as that in ME49 (Additional file 7: Table 5 and Additional file 8: Table S6).

**Figure 6:**
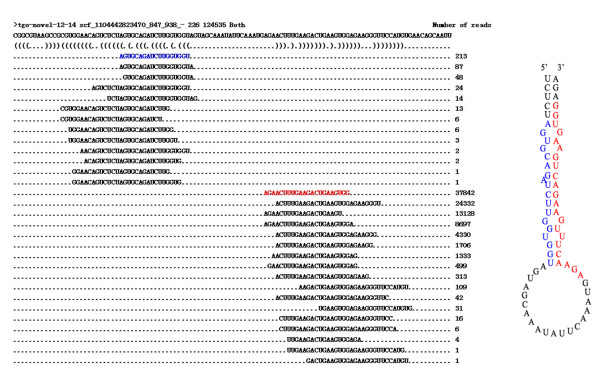
**The sequence and the secondary stem-loop structure of tgo-novel-12-14 identified in* T. gondii. *** Sequences and the number of reads of the mature miRNA and the complementary miR* are represented in red and blue respectively. The predicted structure of the pre-miRNA is represented on the right side.

Previous studies have found that mature miRNAs can be derived from both arms of a pre-miRNA hairpin [44,45]. In this study, we found 178 miRNAs were derived from just one hairpin arm, of which 81 mature miRNAs were located at 3^′^ arm and 97 were located at 5^′^ arm (Figure 6 and Additional file 7: Table S5) of the predicted hairpins. However, one miRNA, tgo-novel-1, the miRNA:miRNA* ratios showed strain-specific pattern. The reads from 3^′^ arm were dominant in ME49 (5^′^/3^′^ read ratio: 443/6212), whereas in RH, the reads number from 5^′^ arm is higher (5^′^/3^′^ read ratio: 9632/2838) (Additional file 7: Table S5).

### Validation of miRNAs expression by Northern blot

**Figure 7:**
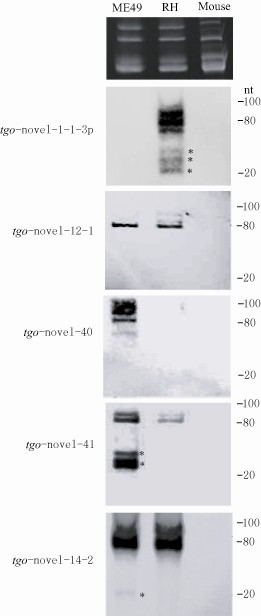
**Characterization of four miRNAs by Northern-blot.** Lanes from left to right are RNAs from ME49 strain (ME49), RH strain (RH) and a mouse. Total RNA isolation from ME49, RH strain and a mouse were visualized by ethidium bromide staining and served as loading controls at the top panel. LNA probes corresponding to tgo-Novel-1-1-3p, tgo-Novel-12-1, tgo-Novel-40, tgo-Novel-41, tgo-Novel-14-2 were used. Probe tgo-Novel-1-1-3p and tgo-Novel-40 only hybridized to the RNAs of RH strain and ME49, respectively. While probes of tgo-Novel-12-1, tgo-Novel-41 and tgo-Novel-14-2 hybridized to RNA of both strains. More hybridization was seen with tgo-Novel-41 probe to ME49. More hybridization was seen with pre-miRNAs except with the probe of tgo-Novel-41. No hybridization was detected with mouse RNA with any probe. Hybridization to mature miRNAs was marked with asterisks.

Five novel miRNAs (tgo-novel-1-1-3p, tgo-novel-12-1, tgo-novel-40, tgo-novel-41, and tgo-novel-14-2) with relatively high abundance identified by sequencing were verified by Northern blot. Specific hybridization with probes of three miRNAs was observed at ≈ 23 nt (Figure 7). In addition, all probes showed hybridization signals to the pre-miRNA transcripts of about 80 nt, except for the precursor of tgo-novel-40, whose signal was detected at ≈ 100 nt. Further, the hybridization signal to the mature miRNAs of tgo-novel-40, tgo-novel-41 and their pre-miRNAs was more intense in ME49 than that in RH, which implying higher expression level in ME49. The expression of tgo-novel-1-1-3p was only detected in RH, which was similar with the results of sequencing analysis. On the contrary, the mature miRNA of tgo-novel-14-2 was only detected in ME49, though the pre-miRNAs with a similar expression level detected in the two strains. The inconsistency with the sequencing data might be due to the slow processing of pre-miRNAs in RH strain. No hybridization was seen with any probe to the mouse miRNAs.

## Discussion

MiRNAs are recognized as critical regulators in gene expression at the post-transcriptional level. Previous studies have found that *T. gondii* possesses a complete RNA silencing pathway which suggests that small noncoding RNAs may play a critical role in the parasite development and its parasitization in the hosts [30]. In this study, the profiles of small RNA populations of the two distinct strains of *T. gondii* were investigated.

The distribution of small RNAs in the two small RNA libraries generated after deep sequencing was compared. A predominant number of small ncRNAs was strain-specific (Figure 1 and Figure 2) and the strain-specific small ncRNA including miRNAs were more in RH strain than in ME49 strain (Figure 1). The difference in miRNA numbers found in the two libraries could be due to the less presentation of ME49 genomic sequences in the databases which may affect small RNA identification. Further, there was a clear difference in the tendency of the length distribution in small RNAs between the two strains. In RH, the 26 nt RNAs represented the predominant species (Figure 3, Additional file 5: Table S3), while in ME49, about 18.92% of the total small RNAs were 21 nt in size, which was the most abundant class. This might be due to the genetic differences between the two parasite strains, which also suggested that the two parasite strains are biologically different. Previous study reported that the structural features of pre-miRNA hairpins might influence the efficiency of Dicer binding and specificity of precursor cleavage, which leads to the length diversity of miRNAs [46,47]. The reasons of strain-specific length distribution of the small ncRNAs between the two strains of *T. gondii* remain vague, further studies are needed to dissect the mechanism in sncRNA processing that may be associated with strain-specific gene regulation.

In total, we identified 17 conserved miRNA and 339 species-specific miRNAs in the two strains of T. gondii, of which 7 miRNAs sequences were reported by Braun et al. in 2010 [30]. Interestingly, about 5% of *T. gondii* miRNAs were categorized as conserved and accounted for less than 1% of the read counts, whereas more than 99% of the remaining miRNAs were recognized as species-specific. This observation supports the earlier finding that *T. gondii* possesses a RNA-associated gene regulation machinery which is phylogenetically diverged from mammals but more similar to plants [30]. Further, let-7 and lin-4, the two most conserved miRNAs in metazoan, were not found in *T. gondii* indicting that the fine-tuning mechanism of miRNAs in *T. gondii* was distinct from other species.

During the process of the biosynthesis of miRNAs, miRNA and miRNA* (or miR*) were generated by enzymatic cleavage of the 70–80 nt precursor hairpin. The functional strand (miRNA) of the small RNA duplex is preferentially loaded into the RISC as the guide strand, while the other strand, the passenger strand (miRNA*), is degraded [44,45]. However, recent studies suggested that mature miRNAs can be generated from both strands of the pre-miRNA duplex [48-50]. In *C. elegans* and related nematodes, it has been reported that the diversity of miRNAs was, at least partially, due to the arm-switching and hairpin shifting [51]. We found that tgo-novel-1 changed the miRNA strand with arm switching of the same hairpin between the two strains of *T. gondii*. In RH strain, the dominant miRNA of tgo-novel-1 was derived from the 3^′^ arm, while in ME49 it seemed that mature miRNA was only derived from the 5^′^ arm. Due to the fact that the sequences of miRNAs derived from the two arms of the same hairpin were complementary, they likely regulate different target sequences. Further dissection of the function of the miRNAs derived from the two arms of the same hairpin might lead to deep understanding of the parasite biology.

## Conclusion

In summary, 17 conserved miRNAs related to 2 metazoan miRNA families and 339 novel miRNAs were identified in the two genetically different strains of *T. gondii.* The majority of miRNAs were species-specific, which supports the finding that *T. gondii* is an evolutionarily diverged organism from other protozoana. The difference in expression abundance of certain miRNAs as well as the arm-switching in pre-miRNA processing leading to different miRNA species in the two parasite strains suggested that there was a fine-tuning mechanism of miRNA biogenesis in distinct strains of *T. gondii*. Understanding the genetic factors that regulate *T. gondii* gene expression could contribute to the development of specific tools to control the transmission of the parasite.

## Competing interests

The authors declare that they have no competing interests.

## Authors’ contributions

JW, XL, BJ, HL, SP, XP, NH, PC, JY performed the experiments. NJ and QC designed and supervised the experiments. JW, XL, NJ and QC wrote the manuscript. All authors read and approved the final version of the manuscript.

## Supplementary Material

Additional file 1 **Figure S1.** The flow chart for detailed analysis of small RNAs isolated from the two strains of *T. gondii*. Click here for file

Additional file 2 **Table S1.** General information of the two libraries. Description: This file contains summary data from high-throughput sequencing of the two small RNA libraries. Click here for file

Additional file 3 **Table S2.** Small RNA classification. Description: This file contains the reads of all small RNA transcripts identified and their relative portions in the library. Click here for file

Additional file 4 **Figure S2.** Statistic analysis of sncRNAs identified in the two libraries. The reads at unique and total levels of the small RNAs in different lengths ranged from 18 to 30 nt were plotted. The difference between the lengths at unique level was significant (p < 0.0001). Click here for file

Additional file 5 **Table S3.** Length distribution of small RNAs identified in the two strains of *T. gondii*. Description: This file contains the reads of small RNAs with different lengths and their relative portions in the library. Click here for file

Additional file 6 **Table S4.** Conserved (common) miRNAs and the genomic loci of the encoding genes identified in the two strains of *T. gondii*. Description: miRNAs with the same sequences could be derived from pre-miRNAs (the sequences may not be completely the same) located at different genomic loci. Their names are indicated with an additional dash-number suffix. Click here for file

Additional file 7 **Table S5.** Novel (unique) miRNAs and the genomic loci of the encoding genes identified in distinct strains of *T. gondii*. Click here for file

Additonal file 8 **Table S6.** Comparative analysis of the expression of novel miRNAs in the two libraries analyzed by software IDEG6 and Edger respectively. Click here for file
