# Supplementary material for: A comparative study of small RNAs in Toxoplasma gondii of distinct genotypes
Source: Parasit Vectors. 2012 Sep 3;5:186. doi: 10.1186/1756-3305-5-186 (PMC3453492; doi:10.1186/1756-3305-5-186)
Supplement: Additional file 2 — Table S1. General information of the two libraries. Description: This file contains summary data from high-throughput sequencing of the two small RNA libraries. [file 1756-3305-5-186-S2.doc]

|  |
| --- |

Additional file 2: Table S1. General information of the two libraries

|  | ME49 | | RH | |
| --- | --- | --- | --- | --- |
|  | Reads | % | Total Reads | % |
| Total Reads | 8738870 | 100.0 | 10759107 | 100.0 |
| Low Quality | 1195457 | 13.7 | 308452 | 2.9 |
| adaptor3 null | 91065 | 1.0 | 404080 | 3.8 |
| insert null | 3087 | 0.0 | 1033762 | 9.6 |
| 5' adaptor contaminants | 88970 | 1.0 | 78207 | 0.7 |
| size < 18 nt | 207311 | 2.4 | 420619 | 3.9 |
| polyA | 3929 | 0.0 | 19233 | 0.2 |
| size >= 18 nt(High-quality Reads) | 7149051 | 81.8 | 8494754 | 79.0 |
