# Supplementary material for: A comparative study of small RNAs in Toxoplasma gondii of distinct genotypes
Source: Parasit Vectors. 2012 Sep 3;5:186. doi: 10.1186/1756-3305-5-186 (PMC3453492; doi:10.1186/1756-3305-5-186)
Supplement: Additional file 3 — Table S2. Small RNA classification. Description: This file contains the reads of all small RNA transcripts identified and their relative portions in the library. [file 1756-3305-5-186-S3.doc]

|  |
| --- |

Additional file 3: Table S2. Small RNA Classification

| Class | Total | | | | ME49 | | | | RH | | | |
| --- | --- | --- | --- | --- | --- | --- | --- | --- | --- | --- | --- | --- |
|  | # of Unique | % | Total sequences | % | # of Unique | % | Total sequences | % | # of Unique | % | Total sequences | % |
| Total Unique Reads | 1083320 |  | 15643805 |  | 247346 |  | 7149051 |  | 867853 |  | 8494754 |  |
| Total Perfect Matched | 184938 | 100 | 9157706 | 100 | 67623 | 100 | 6068932 | 100 | 133106 | 100 | 3088774 | 100 |
| Conserved In Metazoaa | 47 | 0.03 | 1163 | 0.01 | 22 | 0.03 | 854 | 0.01 | 33 | 0.02 | 309 | 0.01 |
| Predicted novel microRNAs^a^ | 11398 | 6.16 | 653089 | 7.13 | 5041 | 7.45 | 489838 | 8.07 | 7623 | 5.73 | 163251 | 5.29 |
| Other Non-coding RNAs^b^ | 7869 | 4.25 | 601680 | 6.57 | 4805 | 7.11 | 453744 | 7.48 | 5402 | 4.06 | 147936 | 4.79 |
| rRNA | 5467 | 2.96 | 531712 | 5.81 | 3631 | 5.37 | 408668 | 6.73 | 3680 | 2.76 | 123044 | 3.98 |
| tRNA | 228 | 0.12 | 3982 | 0.04 | 104 | 0.15 | 828 | 0.01 | 145 | 0.11 | 3154 | 0.10 |
| snoRNA | 28 | 0.02 | 395 | 0.00 | 4 | 0.01 | 19 | 0.00 | 26 | 0.02 | 376 | 0.01 |
| other | 2146 | 1.16 | 65591 | 0.72 | 1066 | 1.58 | 44229 | 0.73 | 1551 | 1.17 | 21362 | 0.69 |
| Unknown | 165624 | 89.56 | 7901774 | 86.29 | 57755 | 85.41 | 5124496 | 84.44 | 120048 | 90.19 | 2777278 | 89.92 |
| ^a^ passed miRcheck. | | | |  |  |  | | | | | | |
| ^b^ Sanger Rfam database release 9.0 ,except microRNA | | | |  |  |  | | | | | | |
