# Supplementary figures and images for: A comparative study of small RNAs in Toxoplasma gondii of distinct genotypes
Source: Parasit Vectors. 2012 Sep 3;5:186. doi: 10.1186/1756-3305-5-186 (PMC3453492; doi:10.1186/1756-3305-5-186)

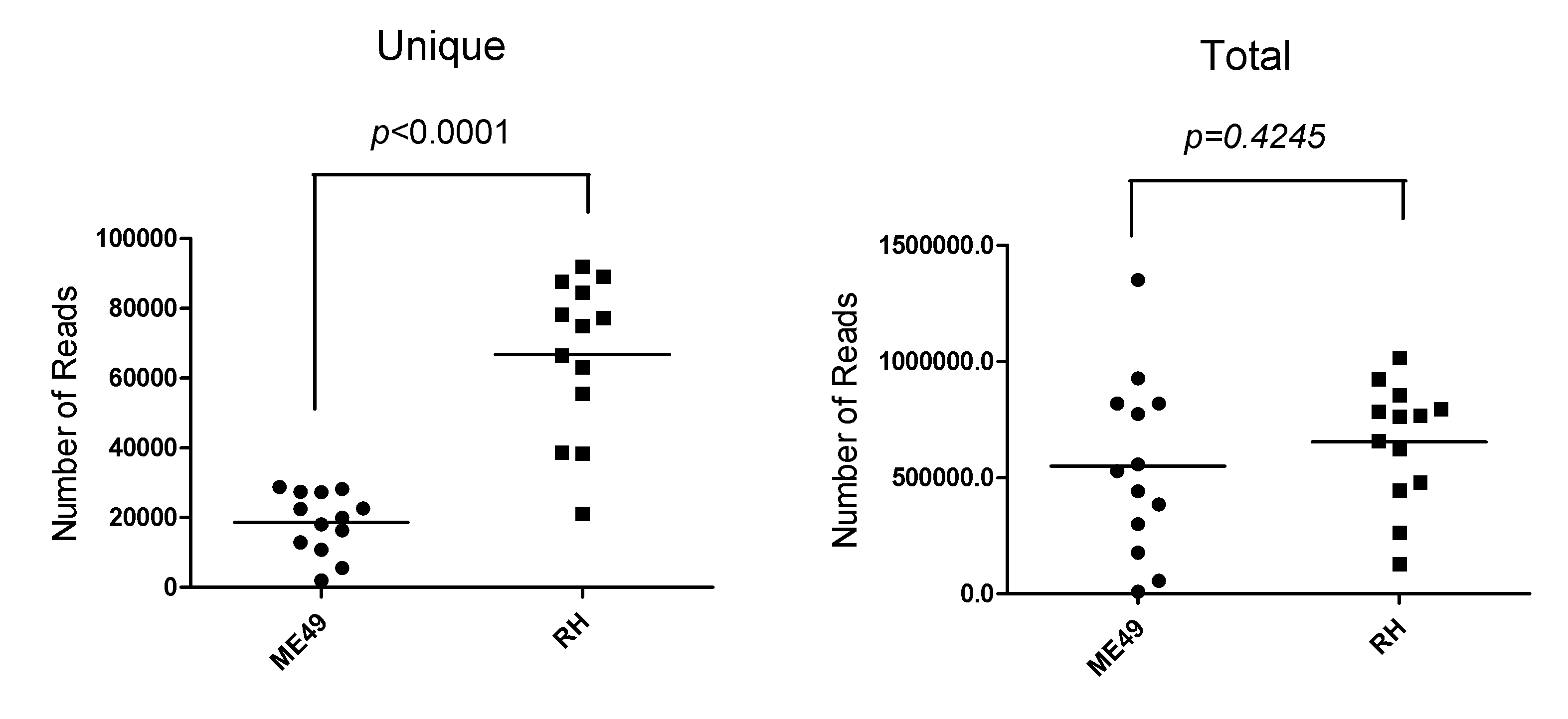

Supplement: Additional file 4 — Figure S2. Statistic analysis of sncRNAs identified in the two libraries. The reads at unique and total levels of the small RNAs in different lengths ranged from 18 to 30 nt were plotted. The difference between the lengths at unique level was significant (p < 0.0001). [file 1756-3305-5-186-S4.tiff]
