# Supplementary material for: A comparative study of small RNAs in Toxoplasma gondii of distinct genotypes
Source: Parasit Vectors. 2012 Sep 3;5:186. doi: 10.1186/1756-3305-5-186 (PMC3453492; doi:10.1186/1756-3305-5-186)
Supplement: Additional file 5 — Table S3. Length distribution of small RNAs identified in the two strains of T. gondii. Description: This file contains the reads of small RNAs with different lengths and their relative portions in the library. [file 1756-3305-5-186-S5.doc]

Additional file 5: Table S3. Length distribution of small RNAs identified in the two strains of *T. gondii*.

| Length | Total Reads | | | | ME49 | | | | RH | | | |
| --- | --- | --- | --- | --- | --- | --- | --- | --- | --- | --- | --- | --- |
|  | Unique | | Total | | Unique | | Total | | Unique | | Total | |
|  | # | % | # | % | # | % | # | % | # | % | # | % |
| 18 | 49753 | 4.61 | 563329 | 3.60 | 12837 | 5.30 | 299698 | 4.20 | 38605 | 4.45 | 263631 | 3.10 |
| 19 | 71317 | 6.61 | 1263380 | 8.08 | 18083 | 7.46 | 818558 | 11.46 | 55531 | 6.40 | 444822 | 5.24 |
| 20 | 86199 | 8.00 | 1430302 | 9.15 | 22430 | 9.25 | 773341 | 10.83 | 66641 | 7.68 | 656961 | 7.73 |
| 21 | 101665 | 9.43 | 1974336 | 12.63 | 27479 | 11.34 | 1350963 | 18.92 | 77319 | 8.91 | 623373 | 7.34 |
| 22 | 114682 | 10.63 | 1688365 | 10.80 | 28818 | 11.89 | 927020 | 12.98 | 89214 | 10.28 | 761345 | 8.96 |
| 23 | 116805 | 10.83 | 1601344 | 10.24 | 28220 | 11.64 | 818194 | 11.46 | 92045 | 10.61 | 783150 | 9.22 |
| 24 | 111815 | 10.37 | 1382687 | 8.84 | 27267 | 11.25 | 528505 | 7.40 | 87812 | 10.12 | 854182 | 10.06 |
| 25 | 104129 | 9.66 | 1150725 | 7.36 | 22588 | 9.32 | 384565 | 5.39 | 84562 | 9.74 | 766160 | 9.02 |
| 26 | 95357 | 8.84 | 1573006 | 10.06 | 19970 | 8.24 | 556891 | 7.80 | 78312 | 9.02 | 1016115 | 11.96 |
| 27 | 88781 | 8.23 | 1235706 | 7.90 | 16389 | 6.76 | 441240 | 6.18 | 75066 | 8.65 | 794466 | 9.35 |
| 28 | 72123 | 6.69 | 1099726 | 7.03 | 10797 | 4.45 | 176315 | 2.47 | 63201 | 7.28 | 923411 | 10.87 |
| 29 | 43053 | 4.00 | 535956 | 3.43 | 5563 | 2.29 | 55987 | 0.78 | 38457 | 4.43 | 479969 | 5.65 |
| 30 | 22701 | 2.11 | 136175 | 0.87 | 1965 | 0.81 | 9006 | 0.13 | 21088 | 2.43 | 127169 | 1.50 |
| In all | 1078380 | 100 | 15635037 | 100 | 242406 | 100 | 7140283 | 100 | 867853 | 100 | 8494754 | 100 |
