# Supplementary material for: A comparative study of small RNAs in Toxoplasma gondii of distinct genotypes
Source: Parasit Vectors. 2012 Sep 3;5:186. doi: 10.1186/1756-3305-5-186 (PMC3453492; doi:10.1186/1756-3305-5-186)
Supplement: Additional file 6 — Table S4. Conserved (common) miRNAs and the genomic loci of the encoding genes identified in the two strains of T. gondii. Description: miRNAs with the same sequences could be derived from pre-miRNAs (the sequences may not be completely the same) located at different genomic loci. Their names are indicated with an additional dash-number suffix. [file 1756-3305-5-186-S6.doc]

Additional file 6: Table S4. Conserved (common) miRNAs identified in the two strains of *T. gondii*

| MicroRNA Name | Gene location | Mature Arm | Position classification | miR*^a^ | Most abundant sequence | Length | Expression^c^ (TPM^b^) | |
| --- | --- | --- | --- | --- | --- | --- | --- | --- |
|  |  |  |  |  |  |  | ME49 | RH |
| miR-574-3 | TGGT1_chrIX_3251181_3251275_+ | 3’ | InterGenic | N | GTGTGTGTGTGTGTGTGTG | 19 | 0 | 4.2 |
| miR-574-7 | TGGT1_chrIb_19848_20009_- | 5’ | Intron | N | GTGTGTGTGTGTGTGTGTG | 19 | 0 | 4.2 |
| miR-574-2 | TGGT1_chrVIIa_1801931_1802033_- | 3’ | InterGenic | N | GTGTGTGTGTGTGTGTGTG | 19 | 0 | 4.2 |
| miR-574-8 | TGME49_chrII_1222390_1222489_- | 3’ | Intron | N | GTGTGTGTGTGTGTGTGTG | 19 | 0 | 4.2 |
| miR-1207-2 | TGME49_chrVIII_2002460_2002564_- | 5’ | UTR | N | TGGCAGGGAGGAACGGAG | 18 | 0 | 0.3 |
| miR-574-10 | TGME49_chrVIII_2383004_2383095_+ | 5’ | Intron | N | GTGTGTGTGTGTGTGTGTG | 19 | 0 | 4.2 |
| miR-574-5 | TGME49_chrVIII_4673365_4673552_+ | 3’ | Intron | N | GTGTGTGTGTGTGTGTGTG | 19 | 0 | 4.2 |
| miR-574-6 | TGME49_chrVIIa_3280763_3280879_- | 3’ | InterGenic | N | GTGTGTGTGTGTGTGTGTG | 19 | 0 | 4.2 |
| miR-574-14 | TGME49_chrVI_1020932_1021166_+ | 3’ | InterGenic | N | TGTGTGTGTGTGTGTGTGTG | 20 | 0.2 | 1.3 |
| miR-574-13 | TGME49_chrXII_2593951_2594121_+ | 3’ | InterGenic | N | GTGTGTGTGTGTGTGTGTG | 19 | 0 | 4.2 |
| miR-574-9 | TGVEG_chrII_1255883_1256048_- | 3’ | Intron | N | GTGTGTGTGTGTGTGTGTG | 19 | 0 | 4.2 |
| miR-574-4 | TGVEG_chrIX_4330458_4330552_+ | 3’ | Intron | N | GTGTGTGTGTGTGTGTGTG | 19 | 0 | 4.2 |
| miR-1207-1 | TGVEG_chrVIII_2002571_2002675_- | 5’ | Intron | N | TGGCAGGGAGGAACGGAG | 18 | 0 | 0.3 |
| miR-574-11 | TGVEG_chrVIII_2382851_2382942_+ | 5’ | InterGenic | N | GTGTGTGTGTGTGTGTGTG | 19 | 0 | 4.2 |
| miR-574-15 | TGVEG_chrVI_2500548_2500782_+ | 3’ | InterGenic | N | TGTGTGTGTGTGTGTGTGTG | 20 | 0.2 | 1.3 |
| miR-574_1 | TGVEG_chrV_741469_741690_- | 5’ | InterGenic | N | GTGTGTGTGTGTGTGTGTG | 19 | 0 | 4.2 |
| miR-574_12 | TGVEG_chrXII_2667187_2667357_+ | 3’ | Intron | N | GTGTGTGTGTGTGTGTGTG | 19 | 0 | 4.2 |

^a^Y indicates that the sequences from both strands of a miRNA* species were found, while N means that only the sequence from one strand of a miRNA* was identified.

^b^The abundance value of each miRNA was normalized to “transcripts per million (TPM)”. If the value after normalization was less than 1, the normalized value was set as 1.

^c^The expression of miRNA was the most abundant sequence of the total counts of unique reads.
