# Supplementary material for: A comparative study of small RNAs in Toxoplasma gondii of distinct genotypes
Source: Parasit Vectors. 2012 Sep 3;5:186. doi: 10.1186/1756-3305-5-186 (PMC3453492; doi:10.1186/1756-3305-5-186)
Supplement: Additional file 7 — Table S5. Novel (unique) miRNAs and the genomic loci of the encoding genes identified in distinct strains of T. gondii. [file 1756-3305-5-186-S7.doc]

Additional file 7: Table S5. Novel (unique) miRNAs identified in distinct strains of *T. gondii*

| MicroRNA Name | Gene location | Mature Arm | Position classification | miR*^a^ | Most abundant sequence | Length | Expression^c^ (TPM^b^) | |
| --- | --- | --- | --- | --- | --- | --- | --- | --- |
|  |  |  |  |  |  |  | ME49 | RH |
| tgo-novel-1-11-3p | scf_1104442822784_858_948_+ | 3’ | InterGenic | Y | GGCGTGTTGCGGACCCGAGCACTGTC | 26 | 442.3 | 9631.3 |
| tgo-novel-1-11-5p | scf_1104442822784_858_948_+ | 5’ | InterGenic | Y | TGACGGAGCTTGGGACTGCTTCATGCT | 27 | 6211.6 | 2737.7 |
| tgo-novel-12-10 | scf_1104442823068_48_135_+ | 3’ | InterGenic | Y | AGAACTTTGAAGACTGAAGTGG | 22 | 6177.5 | 113.6 |
| tgo-novel-40 | scf_1104442825442_837_926_- | 5’ | InterGenic | Y | ACCTGCTAAATAGGATCAGGAA | 22 | 3997.4 | 0.6 |
| tgo-novel-41 | scf_1104442824078_977_1027_+ | 5’ | InterGenic | N | TGCTGGAAGCAGCCAGTCCGCCCT | 24 | 2975.3 | 254.5 |
| tgo-novel-10-1 | TGGT1_chrX_6122614_6122711_- | 5’ | InterGenic | N | TTAGGAGACAAATGCAAGGACTGCTG | 26 | 0 | 21.7 |
| tgo-novel-10-2 | TGME49_chrX_6137888_6137985_- | 5’ | InterGenic | N | TTAGGAGACAAATGCAAGGACTGCTG | 26 | 0 | 21.7 |
| tgo-novel-10-3 | TGVEG_chrX_6204787_6204884_- | 5’ | Intron | N | TTAGGAGACAAATGCAAGGACTGCTG | 26 | 0 | 21.7 |
| tgo-novel-11-1 | TGGT1_chrXI_1836363_1836421_- | 5’ | UTR | N | ACGGAAAGGAGCCGAACCGCGGCATC | 26 | 0 | 1.9 |
| tgo-novel-11-2 | TGME49_chrXI_2216957_2217015_- | 5’ | UTR | N | ACGGAAAGGAGCCGAACCGCGGCATC | 26 | 0 | 1.9 |
| tgo-novel-1-12-3p | scf_1104442822838_803_893_+ | 3’ | InterGenic | Y | GGCGTGTTGCGGACCCGAGCACTGTC | 26 | 442.3 | 9631.3 |
| tgo-novel-1-12-5p | scf_1104442822838_803_893_+ | 5’ | InterGenic | Y | TGACGGAGCTTGGGACTGCTTCATGCT | 27 | 6211.6 | 2737.7 |
| tgo-novel-11-3 | TGVEG_chrXI_2215144_2215202_- | 5’ | InterGenic | N | ACGGAAAGGAGCCGAACCGCGGCATC | 26 | 0 | 1.9 |
| tgo-novel-1-13-3p | scf_1104442822920_548_638_- | 3’ | InterGenic | Y | GGCGTGTTGCGGACCCGAGCACTGTC | 26 | 442.3 | 9631.3 |
| tgo-novel-1-13-5p | scf_1104442822920_548_638_- | 5’ | InterGenic | Y | TGACGGAGCTTGGGACTGCTTCATGCT | 27 | 6211.6 | 2737.7 |
| tgo-novel-1-1-3p | TGGT1_chrIX_4098366_4098456_+ | 3’ | InterGenic | Y | GGCGTGTTGCGGACCCGAGCACTGTC | 26 | 442.3 | 9631.3 |
| tgo-novel-1-14-3p | scf_1104442822940_2_115_+ | 3’ | InterGenic | Y | GGCGTGTTGCGGACCCGAGCACTGTC | 26 | 442.3 | 9631.3 |
| tgo-novel-1-14-5p | scf_1104442822940_2_115_+ | 5’ | InterGenic | Y | TGACGGAGCTTGGGACTGCTTCATGCT | 27 | 6211.6 | 2737.7 |
| tgo-novel-1-15-3p | scf_1104442823068_605_695_+ | 3’ | InterGenic | Y | GGCGTGTTGCGGACCCGAGCACTGTC | 26 | 442.3 | 9631.3 |
| tgo-novel-1-15-5p | scf_1104442823068_605_695_+ | 5’ | InterGenic | Y | TGACGGAGCTTGGGACTGCTTCATGCT | 27 | 6211.6 | 2737.7 |
| tgo-novel-1-1-5p | TGGT1_chrIX_4098366_4098456_+ | 5’ | InterGenic | Y | TGACGGAGCTTGGGACTGCTTCATGCT | 27 | 6211.6 | 2737.7 |
| tgo-novel-1-16-3p | scf_1104442823080_359_449_- | 3’ | InterGenic | Y | GGCGTGTTGCGGACCCGAGCACTGTC | 26 | 442.3 | 9631.3 |
| tgo-novel-1-16-5p | scf_1104442823080_359_449_- | 5’ | InterGenic | Y | TGACGGAGCTTGGGACTGCTTCATGCT | 27 | 6211.6 | 2737.7 |
| tgo-novel-1-17-3p | scf_1104442823086_525_615_- | 3’ | InterGenic | Y | GGCGTGTTGCGGACCCGAGCACTGTC | 26 | 442.3 | 9631.3 |
| tgo-novel-1-17-5p | scf_1104442823086_525_615_- | 5’ | InterGenic | Y | TGACGGAGCTTGGGACTGCTTCATGCT | 27 | 6211.6 | 2737.7 |
| tgo-novel-1-18-3p | scf_1104442823118_824_914_- | 3’ | InterGenic | Y | GGCGTGTTGCGGACCCGAGCACTGTC | 26 | 442.3 | 9631.3 |
| tgo-novel-1-18-5p | scf_1104442823118_824_914_- | 5’ | InterGenic | Y | TGACGGAGCTTGGGACTGCTTCATGCT | 27 | 6211.6 | 2737.7 |
| tgo-novel-1-19-3p | scf_1104442823244_411_501_+ | 3’ | InterGenic | Y | GGCGTGTTGCGGACCCGAGCACTGTC | 26 | 442.3 | 9631.3 |
| tgo-novel-1-19-5p | scf_1104442823244_411_501_+ | 5’ | InterGenic | Y | TGACGGAGCTTGGGACTGCTTCATGCT | 27 | 6211.6 | 2737.7 |
| tgo-novel-1-20-3p | scf_1104442823470_290_380_- | 3’ | InterGenic | Y | GGCGTGTTGCGGACCCGAGCACTGTC | 26 | 442.3 | 9631.3 |
| tgo-novel-1-20-5p | scf_1104442823470_290_380_- | 5’ | InterGenic | Y | TGACGGAGCTTGGGACTGCTTCATGCT | 27 | 6211.6 | 2737.7 |
| tgo-novel-12-11 | scf_1104442823102_547_634_+ | 3’ | InterGenic | Y | AGAACTTTGAAGACTGAAGTGG | 22 | 6177.5 | 113.6 |
| tgo-novel-12-12 | scf_1104442823344_253_340_- | 3’ | InterGenic | Y | AGAACTTTGAAGACTGAAGTGG | 22 | 6177.5 | 113.6 |
| tgo-novel-12-13 | scf_1104442823442_387_474_- | 3’ | InterGenic | Y | AGAACTTTGAAGACTGAAGTGG | 22 | 6177.5 | 113.6 |
| tgo-novel-1-21-3p | scf_1104442823600_4_147_+ | 3’ | InterGenic | Y | GGCGTGTTGCGGACCCGAGCACTGTC | 26 | 442.3 | 9631.3 |
| tgo-novel-12-1 | TGGT1_chrIX_4029435_4029522_- | 3’ | InterGenic | Y | AGAACTTTGAAGACTGAAGTGG | 22 | 6177.5 | 113.6 |
| tgo-novel-12-14 | scf_1104442823470_847_938_- | 3’ | InterGenic | Y | AGAACTTTGAAGACTGAAGTGG | 22 | 6177.5 | 113.6 |
| tgo-novel-12-15 | scf_1104442823510_729_816_+ | 3’ | InterGenic | Y | AGAACTTTGAAGACTGAAGTGG | 22 | 6177.5 | 113.6 |
| tgo-novel-1-21-5p | scf_1104442823600_4_147_+ | 5’ | InterGenic | Y | TGACGGAGCTTGGGACTGCTTCATGCT | 27 | 6211.6 | 2737.7 |
| tgo-novel-12-16 | scf_1104442823646_330_417_- | 3’ | InterGenic | Y | AGAACTTTGAAGACTGAAGTGG | 22 | 6177.5 | 113.6 |
| tgo-novel-12-17 | scf_1104442823784_782_869_- | 3’ | InterGenic | Y | AGAACTTTGAAGACTGAAGTGG | 22 | 6177.5 | 113.6 |
| tgo-novel-12-18 | scf_1104442823842_74_161_+ | 3’ | InterGenic | Y | AGAACTTTGAAGACTGAAGTGG | 22 | 6177.5 | 113.6 |
| tgo-novel-12-19 | scf_1104442823850_207_294_+ | 3’ | InterGenic | Y | AGAACTTTGAAGACTGAAGTGG | 22 | 6177.5 | 113.6 |
| tgo-novel-12-20 | scf_1104442823970_250_337_- | 3’ | InterGenic | Y | AGAACTTTGAAGACTGAAGTGG | 22 | 6177.5 | 113.6 |
| tgo-novel-12-21 | scf_1104442824026_253_340_+ | 3’ | InterGenic | Y | AGAACTTTGAAGACTGAAGTGG | 22 | 6177.5 | 113.6 |
| tgo-novel-12-22 | scf_1104442824336_326_413_- | 3’ | InterGenic | Y | AGAACTTTGAAGACTGAAGTGG | 22 | 6177.5 | 113.6 |
| tgo-novel-12-23 | scf_1104442824374_245_332_+ | 3’ | InterGenic | Y | AGAACTTTGAAGACTGAAGTGG | 22 | 6177.5 | 113.6 |
| tgo-novel-1-22-3p | scf_1104442823664_187_277_+ | 3’ | InterGenic | Y | GGCGTGTTGCGGACCCGAGCACTGTC | 26 | 442.3 | 9631.3 |
| tgo-novel-1-22-5p | scf_1104442823664_187_277_+ | 5’ | InterGenic | Y | TGACGGAGCTTGGGACTGCTTCATGCT | 27 | 6211.6 | 2737.7 |
| tgo-novel-12-2 | scf_1104442822230_583_670_+ | 3’ | InterGenic | Y | AGAACTTTGAAGACTGAAGTGG | 22 | 6177.5 | 113.6 |
| tgo-novel-12-24 | scf_1104442824396_662_749_+ | 3’ | InterGenic | Y | AGAACTTTGAAGACTGAAGTGG | 22 | 6177.5 | 113.6 |
| tgo-novel-12-25 | scf_1104442824604_571_658_+ | 3’ | InterGenic | Y | AGAACTTTGAAGACTGAAGTGG | 22 | 6177.5 | 113.6 |
| tgo-novel-12-26 | scf_1104442824710_46_124_- | 3’ | InterGenic | Y | AGAACTTTGAAGACTGAAGTGG | 22 | 6177.5 | 113.6 |
| tgo-novel-12-27 | scf_1104442824722_838_925_+ | 3’ | InterGenic | Y | AGAACTTTGAAGACTGAAGTGG | 22 | 6177.5 | 113.6 |
| tgo-novel-12-28 | scf_1104442825006_673_760_+ | 3’ | InterGenic | Y | AGAACTTTGAAGACTGAAGTGG | 22 | 6177.5 | 113.6 |
| tgo-novel-12-29 | scf_1104442825228_589_676_- | 3’ | InterGenic | Y | AGAACTTTGAAGACTGAAGTGG | 22 | 6177.5 | 113.6 |
| tgo-novel-12-30 | scf_1104442825276_734_821_- | 3’ | InterGenic | Y | AGAACTTTGAAGACTGAAGTGG | 22 | 6177.5 | 113.6 |
| tgo-novel-12-31 | scf_1104442825390_10_96_- | 3’ | InterGenic | Y | AGAACTTTGAAGACTGAAGTGG | 22 | 6177.5 | 113.6 |
| tgo-novel-12-32 | scf_1104442825416_755_842_- | 3’ | InterGenic | Y | AGAACTTTGAAGACTGAAGTGG | 22 | 6177.5 | 113.6 |
| tgo-novel-12-33 | scf_1104442825530_405_492_+ | 3’ | InterGenic | Y | AGAACTTTGAAGACTGAAGTGG | 22 | 6177.5 | 113.6 |
| tgo-novel-1-23-3p | scf_1104442823666_326_416_+ | 3’ | InterGenic | Y | GGCGTGTTGCGGACCCGAGCACTGTC | 26 | 442.3 | 9631.3 |
| tgo-novel-1-23-5p | scf_1104442823666_326_416_+ | 5’ | InterGenic | Y | TGACGGAGCTTGGGACTGCTTCATGCT | 27 | 6211.6 | 2737.7 |
| tgo-novel-12-3 | scf_1104442822334_832_919_- | 3’ | InterGenic | Y | AGAACTTTGAAGACTGAAGTGG | 22 | 6177.5 | 113.6 |
| tgo-novel-12-34 | scf_1104442825610_188_275_- | 3’ | InterGenic | Y | AGAACTTTGAAGACTGAAGTGG | 22 | 6177.5 | 113.6 |
| tgo-novel-12-35 | scf_1104442825646_648_735_- | 3’ | InterGenic | Y | AGAACTTTGAAGACTGAAGTGG | 22 | 6177.5 | 113.6 |
| tgo-novel-12-36 | scf_1104442825680_366_453_+ | 3’ | InterGenic | Y | AGAACTTTGAAGACTGAAGTGG | 22 | 6177.5 | 113.6 |
| tgo-novel-12-37 | scf_1104442825772_376_463_- | 3’ | InterGenic | Y | AGAACTTTGAAGACTGAAGTGG | 22 | 6177.5 | 113.6 |
| tgo-novel-12-38 | scf_1107000998856_168_255_+ | 3’ | InterGenic | Y | AGAACTTTGAAGACTGAAGTGG | 22 | 6177.5 | 113.6 |
| tgo-novel-12-39 | scf_1107000999372_748_835_- | 3’ | InterGenic | Y | AGAACTTTGAAGACTGAAGTGG | 22 | 6177.5 | 113.6 |
| tgo-novel-1-2-3p | TGGT1_chrIX_4028875_4028965_- | 3’ | InterGenic | Y | GGCGTGTTGCGGACCCGAGCACTGTC | 26 | 442.3 | 9631.3 |
| tgo-novel-1-2-5p | TGGT1_chrIX_4028875_4028965_- | 5’ | InterGenic | Y | TGACGGAGCTTGGGACTGCTTCATGCT | 27 | 6211.6 | 2737.7 |
| tgo-novel-12-40 | scf_1107001000512_351_438_+ | 3’ | InterGenic | Y | AGAACTTTGAAGACTGAAGTGG | 22 | 6177.5 | 113.6 |
| tgo-novel-1-24-3p | scf_1104442823784_222_312_- | 3’ | InterGenic | Y | GGCGTGTTGCGGACCCGAGCACTGTC | 26 | 442.3 | 9631.3 |
| tgo-novel-12-4 | scf_1104442822380_431_518_+ | 3’ | InterGenic | Y | AGAACTTTGAAGACTGAAGTGG | 22 | 6177.5 | 113.6 |
| tgo-novel-1-24-5p | scf_1104442823784_222_312_- | 5’ | InterGenic | Y | TGACGGAGCTTGGGACTGCTTCATGCT | 27 | 6211.6 | 2737.7 |
| tgo-novel-1-25-3p | scf_1104442823804_13_103_+ | 3’ | InterGenic | Y | GGCGTGTTGCGGACCCGAGCACTGTC | 26 | 442.3 | 9631.3 |
| tgo-novel-1-25-5p | scf_1104442823804_13_103_+ | 5’ | InterGenic | Y | TGACGGAGCTTGGGACTGCTTCATGCT | 27 | 6211.6 | 2737.7 |
| tgo-novel-12-5 | scf_1104442822390_442_529_- | 3’ | InterGenic | Y | AGAACTTTGAAGACTGAAGTGG | 22 | 6177.5 | 113.6 |
| tgo-novel-1-26-3p | scf_1104442823842_631_721_+ | 3’ | InterGenic | Y | GGCGTGTTGCGGACCCGAGCACTGTC | 26 | 442.3 | 9631.3 |
| tgo-novel-1-26-5p | scf_1104442823842_631_721_+ | 5’ | InterGenic | Y | TGACGGAGCTTGGGACTGCTTCATGCT | 27 | 6211.6 | 2737.7 |
| tgo-novel-12-6 | scf_1104442822476_495_582_+ | 3’ | InterGenic | Y | AGAACTTTGAAGACTGAAGTGG | 22 | 6177.5 | 113.6 |
| tgo-novel-1-27-3p | scf_1104442823850_764_854_+ | 3’ | InterGenic | Y | GGCGTGTTGCGGACCCGAGCACTGTC | 26 | 442.3 | 9631.3 |
| tgo-novel-1-27-5p | scf_1104442823850_764_854_+ | 5’ | InterGenic | Y | TGACGGAGCTTGGGACTGCTTCATGCT | 27 | 6211.6 | 2737.7 |
| tgo-novel-12-7 | scf_1104442822784_301_388_+ | 3’ | InterGenic | Y | AGAACTTTGAAGACTGAAGTGG | 22 | 6177.5 | 113.6 |
| tgo-novel-1-28-3p | scf_1104442823880_368_458_- | 3’ | InterGenic | Y | GGCGTGTTGCGGACCCGAGCACTGTC | 26 | 442.3 | 9631.3 |
| tgo-novel-1-28-5p | scf_1104442823880_368_458_- | 5’ | InterGenic | Y | TGACGGAGCTTGGGACTGCTTCATGCT | 27 | 6211.6 | 2737.7 |
| tgo-novel-12-8 | scf_1104442822838_246_333_+ | 3’ | InterGenic | Y | AGAACTTTGAAGACTGAAGTGG | 22 | 6177.5 | 113.6 |
| tgo-novel-1-29-3p | scf_1104442824026_810_900_+ | 3’ | InterGenic | Y | GGCGTGTTGCGGACCCGAGCACTGTC | 26 | 442.3 | 9631.3 |
| tgo-novel-12-9 | scf_1104442822966_530_617_+ | 3’ | InterGenic | Y | AGAACTTTGAAGACTGAAGTGG | 22 | 6177.5 | 113.6 |
| tgo-novel-1-29-5p | scf_1104442824026_810_900_+ | 5’ | InterGenic | Y | TGACGGAGCTTGGGACTGCTTCATGCT | 27 | 6211.6 | 2737.7 |
| tgo-novel-1-30-3p | scf_1104442824086_496_586_- | 3’ | InterGenic | Y | GGCGTGTTGCGGACCCGAGCACTGTC | 26 | 442.3 | 9631.3 |
| tgo-novel-1-30-5p | scf_1104442824086_496_586_- | 5’ | InterGenic | Y | TGACGGAGCTTGGGACTGCTTCATGCT | 27 | 6211.6 | 2737.7 |
| tgo-novel-13-1 | TGGT1_chrXII_878306_878395_- | 3’ | InterGenic | N | AACTGAGGAGTCAAGTAGG | 19 | 0 | 7.8 |
| tgo-novel-1-31-3p | scf_1104442824130_249_339_+ | 3’ | InterGenic | Y | GGCGTGTTGCGGACCCGAGCACTGTC | 26 | 442.3 | 9631.3 |
| tgo-novel-1-31-5p | scf_1104442824130_249_339_+ | 5’ | InterGenic | Y | TGACGGAGCTTGGGACTGCTTCATGCT | 27 | 6211.6 | 2737.7 |
| tgo-novel-13-2 | TGME49_chrXII_808309_808398_- | 3’ | InterGenic | N | AACTGAGGAGTCAAGTAGG | 19 | 0 | 7.8 |
| tgo-novel-1-32-3p | scf_1104442824410_374_464_- | 3’ | InterGenic | Y | GGCGTGTTGCGGACCCGAGCACTGTC | 26 | 442.3 | 9631.3 |
| tgo-novel-1-32-5p | scf_1104442824410_374_464_- | 5’ | InterGenic | Y | TGACGGAGCTTGGGACTGCTTCATGCT | 27 | 6211.6 | 2737.7 |
| tgo-novel-13-3 | TGVEG_chrXII_896011_896100_- | 3’ | InterGenic | N | AACTGAGGAGTCAAGTAGG | 19 | 0 | 7.8 |
| tgo-novel-1-33-3p | scf_1104442824442_273_363_+ | 3’ | InterGenic | Y | GGCGTGTTGCGGACCCGAGCACTGTC | 26 | 442.3 | 9631.3 |
| tgo-novel-1-33-5p | scf_1104442824442_273_363_+ | 5’ | InterGenic | Y | TGACGGAGCTTGGGACTGCTTCATGCT | 27 | 6211.6 | 2737.7 |
| tgo-novel-1-3-3p | TGME49_chrIX_5135695_5135785_+ | 3’ | InterGenic | Y | GGCGTGTTGCGGACCCGAGCACTGTC | 26 | 442.3 | 9631.3 |
| tgo-novel-1-3-5p | TGME49_chrIX_5135695_5135785_+ | 5’ | InterGenic | Y | TGACGGAGCTTGGGACTGCTTCATGCT | 27 | 6211.6 | 2737.7 |
| tgo-novel-1-34-3p | scf_1104442824614_458_548_- | 3’ | InterGenic | Y | GGCGTGTTGCGGACCCGAGCACTGTC | 26 | 442.3 | 9631.3 |
| tgo-novel-1-34-5p | scf_1104442824614_458_548_- | 5’ | InterGenic | Y | TGACGGAGCTTGGGACTGCTTCATGCT | 27 | 6211.6 | 2737.7 |
| tgo-novel-1-35-3p | scf_1104442824858_234_324_+ | 3’ | InterGenic | Y | GGCGTGTTGCGGACCCGAGCACTGTC | 26 | 442.3 | 9631.3 |
| tgo-novel-1-35-5p | scf_1104442824858_234_324_+ | 5’ | InterGenic | Y | TGACGGAGCTTGGGACTGCTTCATGCT | 27 | 6211.6 | 2737.7 |
| tgo-novel-1-36-3p | scf_1104442824896_441_531_+ | 3’ | InterGenic | Y | GGCGTGTTGCGGACCCGAGCACTGTC | 26 | 442.3 | 9631.3 |
| tgo-novel-1-36-5p | scf_1104442824896_441_531_+ | 5’ | InterGenic | Y | TGACGGAGCTTGGGACTGCTTCATGCT | 27 | 6211.6 | 2737.7 |
| tgo-novel-1-37-3p | scf_1104442824988_488_578_+ | 3’ | InterGenic | Y | GGCGTGTTGCGGACCCGAGCACTGTC | 26 | 442.3 | 9631.3 |
| tgo-novel-1-37-5p | scf_1104442824988_488_578_+ | 5’ | InterGenic | Y | TGACGGAGCTTGGGACTGCTTCATGCT | 27 | 6211.6 | 2737.7 |
| tgo-novel-1-38-3p | scf_1104442825032_427_517_- | 3’ | InterGenic | Y | GGCGTGTTGCGGACCCGAGCACTGTC | 26 | 442.3 | 9631.3 |
| tgo-novel-1-38-5p | scf_1104442825032_427_517_- | 5’ | InterGenic | Y | TGACGGAGCTTGGGACTGCTTCATGCT | 27 | 6211.6 | 2737.7 |
| tgo-novel-1-39-3p | scf_1104442825214_140_230_+ | 3’ | InterGenic | Y | GGCGTGTTGCGGACCCGAGCACTGTC | 26 | 442.3 | 9631.3 |
| tgo-novel-1-39-5p | scf_1104442825214_140_230_+ | 5’ | InterGenic | Y | TGACGGAGCTTGGGACTGCTTCATGCT | 27 | 6211.6 | 2737.7 |
| tgo-novel-1-40-3p | scf_1104442825276_174_264_- | 3’ | InterGenic | Y | GGCGTGTTGCGGACCCGAGCACTGTC | 26 | 442.3 | 9631.3 |
| tgo-novel-1-40-5p | scf_1104442825276_174_264_- | 5’ | InterGenic | Y | TGACGGAGCTTGGGACTGCTTCATGCT | 27 | 6211.6 | 2737.7 |
| tgo-novel-14-1 | TGGT1_chrVIIb_4650942_4651100_+ | 3’ | InterGenic | N | CGGCGAGAAGGGAAGTGT | 18 | 34.8 | 317.6 |
| tgo-novel-1-41-3p | scf_1104442825378_748_838_- | 3’ | InterGenic | Y | GGCGTGTTGCGGACCCGAGCACTGTC | 26 | 442.3 | 9631.3 |
| tgo-novel-1-41-5p | scf_1104442825378_748_838_- | 5’ | InterGenic | Y | TGACGGAGCTTGGGACTGCTTCATGCT | 27 | 6211.6 | 2737.7 |
| tgo-novel-14-2 | TGME49_chrVIIb_4602245_4602349_+ | 3’ | Intron | N | CGGCGAGAAGGGAAGTGT | 18 | 34.8 | 317.6 |
| tgo-novel-1-42-3p | scf_1104442825416_195_285_- | 3’ | InterGenic | Y | GGCGTGTTGCGGACCCGAGCACTGTC | 26 | 442.3 | 9631.3 |
| tgo-novel-1-42-5p | scf_1104442825416_195_285_- | 5’ | InterGenic | Y | TGACGGAGCTTGGGACTGCTTCATGCT | 27 | 6211.6 | 2737.7 |
| tgo-novel-14-3 | TGVEG_chrVIIb_4640093_4640197_+ | 3’ | InterGenic | N | CGGCGAGAAGGGAAGTGT | 18 | 34.8 | 317.6 |
| tgo-novel-1-43-3p | scf_1104442825498_378_468_+ | 3’ | InterGenic | Y | GGCGTGTTGCGGACCCGAGCACTGTC | 26 | 442.3 | 9631.3 |
| tgo-novel-1-43-5p | scf_1104442825498_378_468_+ | 5’ | InterGenic | Y | TGACGGAGCTTGGGACTGCTTCATGCT | 27 | 6211.6 | 2737.7 |
| tgo-novel-1-4-3p | TGME49_chrIX_5066827_5066917_- | 3’ | InterGenic | Y | GGCGTGTTGCGGACCCGAGCACTGTC | 26 | 442.3 | 9631.3 |
| tgo-novel-1-4-5p | TGME49_chrIX_5066827_5066917_- | 5’ | InterGenic | Y | TGACGGAGCTTGGGACTGCTTCATGCT | 27 | 6211.6 | 2737.7 |
| tgo-novel-1-44-3p | scf_1104442825540_689_779_- | 3’ | InterGenic | Y | GGCGTGTTGCGGACCCGAGCACTGTC | 26 | 442.3 | 9631.3 |
| tgo-novel-1-44-5p | scf_1104442825540_689_779_- | 5’ | InterGenic | Y | TGACGGAGCTTGGGACTGCTTCATGCT | 27 | 6211.6 | 2737.7 |
| tgo-novel-1-45-3p | scf_1104442825618_587_677_- | 3’ | InterGenic | Y | GGCGTGTTGCGGACCCGAGCACTGTC | 26 | 442.3 | 9631.3 |
| tgo-novel-1-45-5p | scf_1104442825618_587_677_- | 5’ | InterGenic | Y | TGACGGAGCTTGGGACTGCTTCATGCT | 27 | 6211.6 | 2737.7 |
| tgo-novel-1-46-3p | scf_1104442825646_88_178_- | 3’ | InterGenic | Y | GGCGTGTTGCGGACCCGAGCACTGTC | 26 | 442.3 | 9631.3 |
| tgo-novel-1-46-5p | scf_1104442825646_88_178_- | 5’ | InterGenic | Y | TGACGGAGCTTGGGACTGCTTCATGCT | 27 | 6211.6 | 2737.7 |
| tgo-novel-1-47-3p | scf_1104442825688_951_1041_- | 3’ | InterGenic | Y | GGCGTGTTGCGGACCCGAGCACTGTC | 26 | 442.3 | 9631.3 |
| tgo-novel-1-47-5p | scf_1104442825688_951_1041_- | 5’ | InterGenic | Y | TGACGGAGCTTGGGACTGCTTCATGCT | 27 | 6211.6 | 2737.7 |
| tgo-novel-1-48-3p | scf_1104442825836_552_642_+ | 3’ | InterGenic | Y | GGCGTGTTGCGGACCCGAGCACTGTC | 26 | 442.3 | 9631.3 |
| tgo-novel-1-48-5p | scf_1104442825836_552_642_+ | 5’ | InterGenic | Y | TGACGGAGCTTGGGACTGCTTCATGCT | 27 | 6211.6 | 2737.7 |
| tgo-novel-1-49-3p | scf_1104442826002_798_888_- | 3’ | InterGenic | Y | GGCGTGTTGCGGACCCGAGCACTGTC | 26 | 442.3 | 9631.3 |
| tgo-novel-1-49-5p | scf_1104442826002_798_888_- | 5’ | InterGenic | Y | TGACGGAGCTTGGGACTGCTTCATGCT | 27 | 6211.6 | 2737.7 |
| tgo-novel-1-50-3p | scf_1107000998856_725_815_+ | 3’ | InterGenic | Y | GGCGTGTTGCGGACCCGAGCACTGTC | 26 | 442.3 | 9631.3 |
| tgo-novel-1-50-5p | scf_1107000998856_725_815_+ | 5’ | InterGenic | Y | TGACGGAGCTTGGGACTGCTTCATGCT | 27 | 6211.6 | 2737.7 |
| tgo-novel-15-1 | TGGT1_chrXI_6188731_6188849_+ | 5’ | InterGenic | N | AAGGAACTTGACAAGCAT | 18 | 34.6 | 1.3 |
| tgo-novel-1-51-3p | scf_1107000999326_191_281_+ | 3’ | InterGenic | Y | GGCGTGTTGCGGACCCGAGCACTGTC | 26 | 442.3 | 9631.3 |
| tgo-novel-1-51-5p | scf_1107000999326_191_281_+ | 5’ | InterGenic | Y | TGACGGAGCTTGGGACTGCTTCATGCT | 27 | 6211.6 | 2737.7 |
| tgo-novel-15-2 | TGME49_chrXI_6192917_6193035_+ | 5’ | InterGenic | N | AAGGAACTTGACAAGCAT | 18 | 34.6 | 1.3 |
| tgo-novel-1-52-3p | scf_1107000999370_746_836_- | 3’ | InterGenic | Y | GGCGTGTTGCGGACCCGAGCACTGTC | 26 | 442.3 | 9631.3 |
| tgo-novel-1-52-5p | scf_1107000999370_746_836_- | 5’ | InterGenic | Y | TGACGGAGCTTGGGACTGCTTCATGCT | 27 | 6211.6 | 2737.7 |
| tgo-novel-15-3 | TGVEG_chrXI_6205625_6205743_+ | 5’ | InterGenic | N | AAGGAACTTGACAAGCAT | 18 | 34.6 | 1.3 |
| tgo-novel-1-53-3p | scf_1107000999372_188_278_- | 3’ | InterGenic | Y | GGCGTGTTGCGGACCCGAGCACTGTC | 26 | 442.3 | 9631.3 |
| tgo-novel-1-53-5p | scf_1107000999372_188_278_- | 5’ | InterGenic | Y | TGACGGAGCTTGGGACTGCTTCATGCT | 27 | 6211.6 | 2737.7 |
| tgo-novel-1-5-3p | TGVEG_chrIX_5111627_5111717_- | 3’ | InterGenic | Y | GGCGTGTTGCGGACCCGAGCACTGTC | 26 | 442.3 | 9631.3 |
| tgo-novel-1-5-5p | TGVEG_chrIX_5111627_5111717_- | 5’ | InterGenic | Y | TGACGGAGCTTGGGACTGCTTCATGCT | 27 | 6211.6 | 2737.7 |
| tgo-novel-1-54-3p | scf_1107001000512_908_998_+ | 3’ | InterGenic | Y | GGCGTGTTGCGGACCCGAGCACTGTC | 26 | 442.3 | 9631.3 |
| tgo-novel-1-54-5p | scf_1107001000512_908_998_+ | 5’ | InterGenic | Y | TGACGGAGCTTGGGACTGCTTCATGCT | 27 | 6211.6 | 2737.7 |
| tgo-novel-16-1 | TGGT1_chrIX_1720134_1720227_- | 5’ | UTR | N | AAGGAAGCATTGGGGCATCAACGT | 24 | 0.8 | 0 |
| tgo-novel-16-2 | TGME49_chrIX_2749964_2750057_- | 5’ | UTR | N | AAGGAAGCATTGGGGCATCAACGT | 24 | 0.8 | 0 |
| tgo-novel-16-3 | TGVEG_chrIX_2797957_2798050_- | 5’ | UTR | N | AAGGAAGCATTGGGGCATCAACGT | 24 | 0.8 | 0 |
| tgo-novel-1-6-3p | scf_1104442822244_359_449_- | 3’ | InterGenic | Y | GGCGTGTTGCGGACCCGAGCACTGTC | 26 | 442.3 | 9631.3 |
| tgo-novel-1-6-5p | scf_1104442822244_359_449_- | 5’ | InterGenic | Y | TGACGGAGCTTGGGACTGCTTCATGCT | 27 | 6211.6 | 2737.7 |
| tgo-novel-17-1 | TGGT1_chrVIIa_989705_989830_- | 5’ | InterGenic | N | TTCGAGACGTCTGCTTTTGCAC | 22 | 4.8 | 0 |
| tgo-novel-17-2 | TGME49_chrVIIa_987079_987204_- | 5’ | InterGenic | N | TTCGAGACGTCTGCTTTTGCAC | 22 | 4.8 | 0 |
| tgo-novel-17-3 | TGVEG_chrVIIa_997589_997714_- | 5’ | InterGenic | N | TTCGAGACGTCTGCTTTTGCAC | 22 | 4.8 | 0 |
| tgo-novel-1-7-3p | scf_1104442822334_272_362_- | 3’ | InterGenic | Y | GGCGTGTTGCGGACCCGAGCACTGTC | 26 | 442.3 | 9631.3 |
| tgo-novel-1-7-5p | scf_1104442822334_272_362_- | 5’ | InterGenic | Y | TGACGGAGCTTGGGACTGCTTCATGCT | 27 | 6211.6 | 2737.7 |
| tgo-novel-18-1 | TGGT1_chrX_1471309_1471358_- | 3’ | UTR | N | AGTAAGAGAAGCGGGGAGAACCAG | 24 | 6.6 | 1 |
| tgo-novel-18-2 | TGME49_chrX_1475709_1475758_- | 3’ | UTR | N | AGTAAGAGAAGCGGGGAGAACCAG | 24 | 6.6 | 1 |
| tgo-novel-18-3 | TGVEG_chrX_1532772_1532821_- | 3’ | Intron | N | AGTAAGAGAAGCGGGGAGAACCAG | 24 | 6.6 | 1 |
| tgo-novel-1-8-3p | scf_1104442822458_307_397_- | 3’ | InterGenic | Y | GGCGTGTTGCGGACCCGAGCACTGTC | 26 | 442.3 | 9631.3 |
| tgo-novel-1-8-5p | scf_1104442822458_307_397_- | 5’ | InterGenic | Y | TGACGGAGCTTGGGACTGCTTCATGCT | 27 | 6211.6 | 2737.7 |
| tgo-novel-19-1 | TGME49_chrIX_387387_387493_+ | 3’ | InterGenic | N | TGGAGCAACTGTGGAGGCGAC | 21 | 0.3 | 1.6 |
| tgo-novel-19-2 | TGVEG_chrIX_930676_930782_+ | 3’ | Intron | N | TGGAGCAACTGTGGAGGCGAC | 21 | 0.3 | 1.6 |
| tgo-novel-1-9-3p | scf_1104442822574_329_419_- | 3’ | InterGenic | Y | GGCGTGTTGCGGACCCGAGCACTGTC | 26 | 442.3 | 9631.3 |
| tgo-novel-1-9-5p | scf_1104442822574_329_419_- | 5’ | InterGenic | Y | TGACGGAGCTTGGGACTGCTTCATGCT | 27 | 6211.6 | 2737.7 |
| tgo-novel-20-1 | TGGT1_chrVIII_5812212_5812328_- | 5’ | InterGenic | N | AAGGCGACGTGGAGAGGGG | 19 | 0 | 1.6 |
| tgo-novel-20-2 | TGVEG_chrVIII_5812574_5812690_- | 5’ | Intron | N | AAGGCGACGTGGAGAGGGG | 19 | 0 | 1.6 |
| tgo-novel-21-1 | TGME49_chrV_367945_367993_- | 5’ | UTR | N | ACCACGGGAATGGCACAAATGCGG | 24 | 1.5 | 0 |
| tgo-novel-21-2 | TGVEG_chrV_201120_201168_+ | 5’ | InterGenic | N | ACCACGGGAATGGCACAAATGCGG | 24 | 1.5 | 0 |
| tgo-novel-2-1 | TGGT1_chrX_6121119_6121191_- | 5’ | InterGenic | Y | AGTGCCTCGGTTGTTGTTC | 19 | 0 | 15.9 |
| tgo-novel-22-1 | TGME49_chrIX_3433047_3433212_- | 3’ | Intron | N | AGAAGAAAGACGTCGAGATCGAGT | 24 | 1 | 0 |
| tgo-novel-22-2 | TGVEG_chrIX_3479480_3479645_- | 3’ | Intron | N | AGAAGAAAGACGTCGAGATCGAGT | 24 | 1 | 0 |
| tgo-novel-2-2 | TGME49_chrX_6136356_6136428_- | 5’ | InterGenic | Y | AGTGCCTCGGTTGTTGTTC | 19 | 0 | 15.9 |
| tgo-novel-23-1 | TGME49_chrIX_5141246_5141291_+ | 5’ | InterGenic | N | GTTGGATATCCTGCGCTGCTTCCAA | 25 | 0 | 2.3 |
| tgo-novel-23-10 | scf_1104442824080_125_170_- | 5’ | InterGenic | N | GTTGGATATCCTGCGCTGCTTCCAA | 25 | 0 | 2.3 |
| tgo-novel-23-11 | scf_1104442824198_139_184_+ | 5’ | InterGenic | N | GTTGGATATCCTGCGCTGCTTCCAA | 25 | 0 | 2.3 |
| tgo-novel-23-12 | scf_1104442824260_286_331_+ | 5’ | InterGenic | N | GTTGGATATCCTGCGCTGCTTCCAA | 25 | 0 | 2.3 |
| tgo-novel-23-13 | scf_1104442824280_627_672_+ | 5’ | InterGenic | N | GTTGGATATCCTGCGCTGCTTCCAA | 25 | 0 | 2.3 |
| tgo-novel-23-14 | scf_1104442824398_219_264_- | 5’ | InterGenic | N | GTTGGATATCCTGCGCTGCTTCCAA | 25 | 0 | 2.3 |
| tgo-novel-23-15 | scf_1104442824436_883_927_+ | 5’ | InterGenic | N | GTTGGATATCCTGCGCTGCTTCCAA | 25 | 0 | 2.3 |
| tgo-novel-23-16 | scf_1104442824566_358_403_+ | 5’ | InterGenic | N | GTTGGATATCCTGCGCTGCTTCCAA | 25 | 0 | 2.3 |
| tgo-novel-23-17 | scf_1104442824790_545_590_- | 5’ | InterGenic | N | GTTGGATATCCTGCGCTGCTTCCAA | 25 | 0 | 2.3 |
| tgo-novel-23-18 | scf_1104442824836_714_759_- | 5’ | InterGenic | N | GTTGGATATCCTGCGCTGCTTCCAA | 25 | 0 | 2.3 |
| tgo-novel-23-19 | scf_1104442825164_674_719_- | 5’ | InterGenic | N | GTTGGATATCCTGCGCTGCTTCCAA | 25 | 0 | 2.3 |
| tgo-novel-23-2 | scf_1104442822416_574_619_+ | 5’ | InterGenic | N | GTTGGATATCCTGCGCTGCTTCCAA | 25 | 0 | 2.3 |
| tgo-novel-23-20 | scf_1104442825394_323_368_+ | 5’ | InterGenic | N | GTTGGATATCCTGCGCTGCTTCCAA | 25 | 0 | 2.3 |
| tgo-novel-23-21 | scf_1104442825426_775_819_+ | 5’ | InterGenic | N | GTTGGATATCCTGCGCTGCTTCCAA | 25 | 0 | 2.3 |
| tgo-novel-23-22 | scf_1104442825442_268_313_- | 5’ | InterGenic | N | GTTGGATATCCTGCGCTGCTTCCAA | 25 | 0 | 2.3 |
| tgo-novel-23-23 | scf_1104442825522_459_504_+ | 5’ | InterGenic | N | GTTGGATATCCTGCGCTGCTTCCAA | 25 | 0 | 2.3 |
| tgo-novel-23-24 | scf_1104442825588_474_519_+ | 5’ | InterGenic | N | GTTGGATATCCTGCGCTGCTTCCAA | 25 | 0 | 2.3 |
| tgo-novel-23-25 | scf_1104442825926_530_575_+ | 5’ | InterGenic | N | GTTGGATATCCTGCGCTGCTTCCAA | 25 | 0 | 2.3 |
| tgo-novel-23-26 | scf_1107001000308_6_51_- | 5’ | InterGenic | N | GTTGGATATCCTGCGCTGCTTCCAA | 25 | 0 | 2.3 |
| tgo-novel-23-3 | scf_1104442822568_162_207_+ | 5’ | InterGenic | N | GTTGGATATCCTGCGCTGCTTCCAA | 25 | 0 | 2.3 |
| tgo-novel-2-3 | TGVEG_chrX_6203254_6203326_- | 5’ | Intron | Y | AGTGCCTCGGTTGTTGTTC | 19 | 0 | 15.9 |
| tgo-novel-23-4 | scf_1104442822816_425_470_- | 5’ | InterGenic | N | GTTGGATATCCTGCGCTGCTTCCAA | 25 | 0 | 2.3 |
| tgo-novel-23-5 | scf_1104442823006_400_445_+ | 5’ | InterGenic | N | GTTGGATATCCTGCGCTGCTTCCAA | 25 | 0 | 2.3 |
| tgo-novel-23-6 | scf_1104442823392_538_583_+ | 5’ | InterGenic | N | GTTGGATATCCTGCGCTGCTTCCAA | 25 | 0 | 2.3 |
| tgo-novel-23-7 | scf_1104442823694_62_107_+ | 5’ | InterGenic | N | GTTGGATATCCTGCGCTGCTTCCAA | 25 | 0 | 2.3 |
| tgo-novel-23-8 | scf_1104442823708_87_132_+ | 5’ | InterGenic | N | GTTGGATATCCTGCGCTGCTTCCAA | 25 | 0 | 2.3 |
| tgo-novel-23-9 | scf_1104442823956_31_76_- | 5’ | InterGenic | N | GTTGGATATCCTGCGCTGCTTCCAA | 25 | 0 | 2.3 |
| tgo-novel-24-1 | DS984804_1052_1115_- | 5’ | InterGenic | N | TGGATTGGTATGTTCTTCCTT | 21 | 0 | 1.6 |
| tgo-novel-24-2 | scf_1104442825590_878_941_+ | 5’ | InterGenic | N | TGGATTGGTATGTTCTTCCTT | 21 | 0 | 1.6 |
| tgo-novel-25-1 | scf_1104442823664_644_768_+ | 3’ | InterGenic | N | ACTCTTAATGTCGTTTTACTT | 21 | 45.6 | 47.6 |
| tgo-novel-25-2 | scf_1104442824086_6_129_- | 3’ | InterGenic | N | ACTCTTAATGTCGTTTTACTT | 21 | 45.6 | 47.6 |
| tgo-novel-26-1 | TGME49_chrVIIa_987234_987297_- | 5’ | InterGenic | N | GCAGATGTGGGAACAAGTGCACCC | 24 | 11.7 | 0 |
| tgo-novel-26-2 | TGVEG_chrVIIa_997744_997807_- | 5’ | InterGenic | N | GCAGATGTGGGAACAAGTGCACCC | 24 | 11.7 | 0 |
| tgo-novel-27-1 | TGGT1_chrX_238488_238616_+ | 5’ | Intron | N | TGCGGGAGCTCTTCTGGATCGAC | 23 | 0 | 1.9 |
| tgo-novel-27-2 | TGVEG_chrX_300214_300342_+ | 5’ | InterGenic | N | TGCGGGAGCTCTTCTGGATCGAC | 23 | 0 | 1.9 |
| tgo-novel-28-1-3p | TGME49_chrVIII_1264480_1264659_+ | 3’ | UTR | Y | GCCGCGGCGATTCCTCGACT | 20 | 0.8 | 0 |
| tgo-novel-28-1-5p | TGME49_chrVIII_1264480_1264659_+ | 5’ | UTR | Y | AAAGTCGACGTTTCCTCGCGGG | 22 | 0.3 | 0.3 |
| tgo-novel-28-2-3p | TGVEG_chrVIII_1264186_1264365_+ | 3’ | InterGenic | Y | GCCGCGGCGATTCCTCGACT | 20 | 0.8 | 0 |
| tgo-novel-28-2-5p | TGVEG_chrVIII_1264186_1264365_+ | 5’ | InterGenic | Y | AAAGTCGACGTTTCCTCGCGGG | 22 | 0.3 | 0.3 |
| tgo-novel-29 | DS984804_1788_1952_+ | 5’ | InterGenic | N | CAGTGGTTAGAGCTTTCGGCTGT | 23 | 0 | 12 |
| tgo-novel-30 | TGME49_chrVIIa_2011982_2012070_+ | 5’ | InterGenic | N | AGCTCAGCGTAAGGGTCGCGGAAGC | 25 | 1 | 0 |
| tgo-novel-31 | TGGT1_chrXI_568812_569006_- | 5’ | InterGenic | N | TTCGCGAGGACGGCCTGTGGA | 21 | 0 | 2.3 |
| tgo-novel-3-1-3p | TGGT1_chrIX_3466583_3466663_+ | 3’ | Intron | Y | AAGCGACCATACTCTCTG | 18 | 0 | 1 |
| tgo-novel-3-1-5p | TGGT1_chrIX_3466583_3466663_+ | 5’ | Intron | Y | GAGAATGATGATCCTTTA | 18 | 0 | 1.6 |
| tgo-novel-32 | TGVEG_chrVIIb_1074188_1074322_+ | 5’ | InterGenic | N | AGTCGATGTGGGTCTGGGAAAAC | 23 | 0 | 3.9 |
| tgo-novel-3-2-3p | TGME49_chrIX_4500751_4500831_+ | 3’ | Intron | Y | AAGCGACCATACTCTCTG | 18 | 0 | 1 |
| tgo-novel-3-2-5p | TGME49_chrIX_4500751_4500831_+ | 5’ | Intron | Y | GAGAATGATGATCCTTTA | 18 | 0 | 1.6 |
| tgo-novel-33 | TGGT1_chrXII_5895104_5895286_+ | 5’ | Intron | N | GTCGGGGAGCAGCTCGGGGAGAAG | 24 | 0 | 2.6 |
| tgo-novel-3-3-3p | TGVEG_chrIX_4548654_4548734_+ | 3’ | Intron | Y | AAGCGACCATACTCTCTG | 18 | 0 | 1 |
| tgo-novel-3-3-5p | TGVEG_chrIX_4548654_4548734_+ | 5’ | Intron | Y | GAGAATGATGATCCTTTA | 18 | 0 | 1.6 |
| tgo-novel-34-10-3p | scf_1104442824260_445_523_+ | 3’ | InterGenic | Y | TCTCTCTCGATGTGCTTTCAGA | 22 | 0 | 0.6 |
| tgo-novel-34-10-5p | scf_1104442824260_445_523_+ | 5’ | InterGenic | Y | AAATCTGAGGAACATTTGAGAGAGAG | 26 | 0 | 3.9 |
| tgo-novel-34-11-3p | scf_1104442824280_786_864_+ | 3’ | InterGenic | Y | TCTCTCTCGATGTGCTTTCAGA | 22 | 0 | 0.6 |
| tgo-novel-34-11-5p | scf_1104442824280_786_864_+ | 5’ | InterGenic | Y | AAATCTGAGGAACATTTGAGAGAGAG | 26 | 0 | 3.9 |
| tgo-novel-34-12-3p | scf_1104442824344_734_812_- | 3’ | InterGenic | Y | TCTCTCTCGATGTGCTTTCAGA | 22 | 0 | 0.6 |
| tgo-novel-34-12-5p | scf_1104442824344_734_812_- | 5’ | InterGenic | Y | AAATCTGAGGAACATTTGAGAGAGAG | 26 | 0 | 3.9 |
| tgo-novel-34-13-3p | scf_1104442824398_27_105_- | 3’ | InterGenic | Y | TCTCTCTCGATGTGCTTTCAGA | 22 | 0 | 0.6 |
| tgo-novel-34-13-5p | scf_1104442824398_27_105_- | 5’ | InterGenic | Y | AAATCTGAGGAACATTTGAGAGAGAG | 26 | 0 | 3.9 |
| tgo-novel-34-1-3p | scf_1104442822416_733_811_+ | 3’ | InterGenic | Y | TCTCTCTCGATGTGCTTTCAGA | 22 | 0 | 0.6 |
| tgo-novel-34-14-3p | scf_1104442824566_517_595_+ | 3’ | InterGenic | Y | TCTCTCTCGATGTGCTTTCAGA | 22 | 0 | 0.6 |
| tgo-novel-34-14-5p | scf_1104442824566_517_595_+ | 5’ | InterGenic | Y | AAATCTGAGGAACATTTGAGAGAGAG | 26 | 0 | 3.9 |
| tgo-novel-34-15-3p | scf_1104442824790_353_431_- | 3’ | InterGenic | Y | TCTCTCTCGATGTGCTTTCAGA | 22 | 0 | 0.6 |
| tgo-novel-34-15-5p | scf_1104442824790_353_431_- | 5’ | InterGenic | Y | AAATCTGAGGAACATTTGAGAGAGAG | 26 | 0 | 3.9 |
| tgo-novel-34-1-5p | scf_1104442822416_733_811_+ | 5’ | InterGenic | Y | AAATCTGAGGAACATTTGAGAGAGAG | 26 | 0 | 3.9 |
| tgo-novel-34-16-3p | scf_1104442824836_522_600_- | 3’ | InterGenic | Y | TCTCTCTCGATGTGCTTTCAGA | 22 | 0 | 0.6 |
| tgo-novel-34-16-5p | scf_1104442824836_522_600_- | 5’ | InterGenic | Y | AAATCTGAGGAACATTTGAGAGAGAG | 26 | 0 | 3.9 |
| tgo-novel-34-17-3p | scf_1104442824926_12_89_+ | 3’ | InterGenic | Y | TCTCTCTCGATGTGCTTTCAGA | 22 | 0 | 0.6 |
| tgo-novel-34-17-5p | scf_1104442824926_12_89_+ | 5’ | InterGenic | Y | AAATCTGAGGAACATTTGAGAGAGAG | 26 | 0 | 3.9 |
| tgo-novel-34-18-3p | scf_1104442825022_174_252_+ | 3’ | InterGenic | Y | TCTCTCTCGATGTGCTTTCAGA | 22 | 0 | 0.6 |
| tgo-novel-34-18-5p | scf_1104442825022_174_252_+ | 5’ | InterGenic | Y | AAATCTGAGGAACATTTGAGAGAGAG | 26 | 0 | 3.9 |
| tgo-novel-34-19-3p | scf_1104442825164_482_560_- | 3’ | InterGenic | Y | TCTCTCTCGATGTGCTTTCAGA | 22 | 0 | 0.6 |
| tgo-novel-34-19-5p | scf_1104442825164_482_560_- | 5’ | InterGenic | Y | AAATCTGAGGAACATTTGAGAGAGAG | 26 | 0 | 3.9 |
| tgo-novel-34-20-3p | scf_1104442825394_482_560_+ | 3’ | InterGenic | Y | TCTCTCTCGATGTGCTTTCAGA | 22 | 0 | 0.6 |
| tgo-novel-34-20-5p | scf_1104442825394_482_560_+ | 5’ | InterGenic | Y | AAATCTGAGGAACATTTGAGAGAGAG | 26 | 0 | 3.9 |
| tgo-novel-34-21-3p | scf_1104442825442_76_154_- | 3’ | InterGenic | Y | TCTCTCTCGATGTGCTTTCAGA | 22 | 0 | 0.6 |
| tgo-novel-34-21-5p | scf_1104442825442_76_154_- | 5’ | InterGenic | Y | AAATCTGAGGAACATTTGAGAGAGAG | 26 | 0 | 3.9 |
| tgo-novel-34-22-3p | scf_1104442825522_618_696_+ | 3’ | InterGenic | Y | TCTCTCTCGATGTGCTTTCAGA | 22 | 0 | 0.6 |
| tgo-novel-34-22-5p | scf_1104442825522_618_696_+ | 5’ | InterGenic | Y | AAATCTGAGGAACATTTGAGAGAGAG | 26 | 0 | 3.9 |
| tgo-novel-34-23-3p | scf_1104442825588_633_711_+ | 3’ | InterGenic | Y | TCTCTCTCGATGTGCTTTCAGA | 22 | 0 | 0.6 |
| tgo-novel-34-23-5p | scf_1104442825588_633_711_+ | 5’ | InterGenic | Y | AAATCTGAGGAACATTTGAGAGAGAG | 26 | 0 | 3.9 |
| tgo-novel-34-2-3p | scf_1104442822568_321_399_+ | 3’ | InterGenic | Y | TCTCTCTCGATGTGCTTTCAGA | 22 | 0 | 0.6 |
| tgo-novel-34-24-3p | scf_1104442825656_140_218_+ | 3’ | InterGenic | Y | TCTCTCTCGATGTGCTTTCAGA | 22 | 0 | 0.6 |
| tgo-novel-34-24-5p | scf_1104442825656_140_218_+ | 5’ | InterGenic | Y | AAATCTGAGGAACATTTGAGAGAGAG | 26 | 0 | 3.9 |
| tgo-novel-34-25-3p | scf_1104442825926_689_767_+ | 3’ | InterGenic | Y | TCTCTCTCGATGTGCTTTCAGA | 22 | 0 | 0.6 |
| tgo-novel-34-25-5p | scf_1104442825926_689_767_+ | 5’ | InterGenic | Y | AAATCTGAGGAACATTTGAGAGAGAG | 26 | 0 | 3.9 |
| tgo-novel-34-2-5p | scf_1104442822568_321_399_+ | 5’ | InterGenic | Y | AAATCTGAGGAACATTTGAGAGAGAG | 26 | 0 | 3.9 |
| tgo-novel-34-26-3p | scf_1104442825952_314_392_+ | 3’ | InterGenic | Y | TCTCTCTCGATGTGCTTTCAGA | 22 | 0 | 0.6 |
| tgo-novel-34-26-5p | scf_1104442825952_314_392_+ | 5’ | InterGenic | Y | AAATCTGAGGAACATTTGAGAGAGAG | 26 | 0 | 3.9 |
| tgo-novel-34-3-3p | scf_1104442822816_233_311_- | 3’ | InterGenic | Y | TCTCTCTCGATGTGCTTTCAGA | 22 | 0 | 0.6 |
| tgo-novel-34-3-5p | scf_1104442822816_233_311_- | 5’ | InterGenic | Y | AAATCTGAGGAACATTTGAGAGAGAG | 26 | 0 | 3.9 |
| tgo-novel-34-4-3p | scf_1104442823006_559_637_+ | 3’ | InterGenic | Y | TCTCTCTCGATGTGCTTTCAGA | 22 | 0 | 0.6 |
| tgo-novel-34-4-5p | scf_1104442823006_559_637_+ | 5’ | InterGenic | Y | AAATCTGAGGAACATTTGAGAGAGAG | 26 | 0 | 3.9 |
| tgo-novel-34-5-3p | scf_1104442823392_697_775_+ | 3’ | InterGenic | Y | TCTCTCTCGATGTGCTTTCAGA | 22 | 0 | 0.6 |
| tgo-novel-34-5-5p | scf_1104442823392_697_775_+ | 5’ | InterGenic | Y | AAATCTGAGGAACATTTGAGAGAGAG | 26 | 0 | 3.9 |
| tgo-novel-34-6-3p | scf_1104442823694_221_299_+ | 3’ | InterGenic | Y | TCTCTCTCGATGTGCTTTCAGA | 22 | 0 | 0.6 |
| tgo-novel-34-6-5p | scf_1104442823694_221_299_+ | 5’ | InterGenic | Y | AAATCTGAGGAACATTTGAGAGAGAG | 26 | 0 | 3.9 |
| tgo-novel-34-7-3p | scf_1104442823708_246_324_+ | 3’ | InterGenic | Y | TCTCTCTCGATGTGCTTTCAGA | 22 | 0 | 0.6 |
| tgo-novel-34-7-5p | scf_1104442823708_246_324_+ | 5’ | InterGenic | Y | AAATCTGAGGAACATTTGAGAGAGAG | 26 | 0 | 3.9 |
| tgo-novel-34-8-3p | scf_1104442824076_688_766_- | 3’ | InterGenic | Y | TCTCTCTCGATGTGCTTTCAGA | 22 | 0 | 0.6 |
| tgo-novel-34-8-5p | scf_1104442824076_688_766_- | 5’ | InterGenic | Y | AAATCTGAGGAACATTTGAGAGAGAG | 26 | 0 | 3.9 |
| tgo-novel-34-9-3p | scf_1104442824198_298_376_+ | 3’ | InterGenic | Y | TCTCTCTCGATGTGCTTTCAGA | 22 | 0 | 0.6 |
| tgo-novel-34-9-5p | scf_1104442824198_298_376_+ | 5’ | InterGenic | Y | AAATCTGAGGAACATTTGAGAGAGAG | 26 | 0 | 3.9 |
| tgo-novel-35 | scf_1104442825588_15_82_+ | 3’ | InterGenic | Y | ATGCATCGTGATGGGGAT | 18 | 111.2 | 60.9 |
| tgo-novel-36 | TGME49_chrVIIb_846052_846136_- | 3’ | UTR | N | TCGGCCGAGAGCCTGAACGCGAACG | 25 | 0 | 2.3 |
| tgo-novel-37 | TGME49_chrXII_1356174_1356365_- | 5’ | InterGenic | N | AACGGACGCCGCGGGAGAGAGGAG | 24 | 0 | 2.3 |
| tgo-novel-38 | DS984804_1670_1906_- | 3’ | InterGenic | N | ATATAGCTCAGGAGGTAGA | 19 | 0.2 | 6.2 |
| tgo-novel-39 | TGGT1_chrXI_5550231_5550328_+ | 3’ | InterGenic | N | GGAGCAACGTCCTTCTGTGGAGGTGT | 26 | 0 | 2.3 |
| tgo-novel-4-1 | TGGT1_chrX_1472813_1472885_- | 3’ | Intron | N | CGGAGCGTCAGTTGGAAGTTG | 21 | 0 | 9.4 |
| tgo-novel-42 | TGVEG_chrX_2396029_2396129_- | 3’ | Intron | N | TCAGGGACGAAGGGGAAGGCAGCGAC | 26 | 0 | 1.3 |
| tgo-novel-4-2 | TGME49_chrX_1477207_1477279_- | 3’ | Intron | N | CGGAGCGTCAGTTGGAAGTTG | 21 | 0 | 9.4 |
| tgo-novel-43 | scf_1107000999372_881_918_- | 3’ | InterGenic | N | TTGTAGCAATGAGTAGGA | 18 | 17 | 6.8 |
| tgo-novel-4-3 | TGVEG_chrX_1534290_1534362_- | 3’ | Intron | N | CGGAGCGTCAGTTGGAAGTTG | 21 | 0 | 9.4 |
| tgo-novel-44 | TGME49_chrX_5838934_5839135_+ | 5’ | Intron | N | TGCGGTTTCTCTGGGGAGGACGG | 23 | 0 | 3.9 |
| tgo-novel-45-1-3p | TGME49_chrVIII_1265548_1265694_+ | 3’ | InterGenic | Y | ATTGAGGCATTTGTCTGACG | 20 | 0.2 | 1.3 |
| tgo-novel-45-1-5p | TGME49_chrVIII_1265548_1265694_+ | 5’ | InterGenic | Y | CCAGAGTGATGACCTTTGA | 19 | 0 | 1 |
| tgo-novel-45-2-3p | TGME49_chrVIII_1266194_1266320_+ | 3’ | InterGenic | Y | ATTGAGGCATTTGTCTGACG | 20 | 0.2 | 1.3 |
| tgo-novel-45-2-5p | TGME49_chrVIII_1266194_1266320_+ | 5’ | InterGenic | Y | CCAGAGTGATGACCTTTGA | 19 | 0 | 1 |
| tgo-novel-45-3-3p | TGVEG_chrVIII_1265272_1265406_+ | 3’ | InterGenic | Y | ATTGAGGCATTTGTCTGACG | 20 | 0.2 | 1.3 |
| tgo-novel-45-3-5p | TGVEG_chrVIII_1265272_1265406_+ | 5’ | InterGenic | Y | CCAGAGTGATGACCTTTGA | 19 | 0 | 1 |
| tgo-novel-45-4-3p | TGVEG_chrVIII_1265912_1266038_+ | 3’ | InterGenic | Y | ATTGAGGCATTTGTCTGACG | 20 | 0.2 | 1.3 |
| tgo-novel-45-4-5p | TGVEG_chrVIII_1265912_1266038_+ | 5’ | InterGenic | Y | CCAGAGTGATGACCTTTGA | 19 | 0 | 1 |
| tgo-novel-46 | DS984821_5410_5568_+ | 5’ | InterGenic | N | GGTAAAGGAATATATGAA | 18 | 0 | 2.6 |
| tgo-novel-47-3p | DS984804_8442_8589_+ | 3’ | InterGenic | Y | GTGGATATGATTTTGAAGAT | 20 | 0 | 1 |
| tgo-novel-47-5p | DS984804_8442_8589_+ | 5’ | InterGenic | Y | CAAGATATTATAGACTCAGAATTTG | 25 | 0 | 9.1 |
| tgo-novel-48 | TGGT1_chrXI_3707505_3707667_- | 3’ | InterGenic | N | GACATGTGAAGAGTCGGACC | 20 | 0.2 | 2.6 |
| tgo-novel-49 | scf_1107000999778_3733_3868_- | 5’ | UTR | N | AGAGAACATCGGAGTCTGCTT | 21 | 0.8 | 0 |
| tgo-novel-50 | scf_1107001000004_76418_76480_+ | 5’ | InterGenic | N | ATATGGATTACTGTTATCACTC | 22 | 1 | 1 |
| tgo-novel-51 | TGGT1_chrVIII_3146311_3146435_+ | 5’ | InterGenic | N | AAGCGCCATCGGGGCTGAGCTCGCCGGC | 28 | 0 | 6.8 |
| tgo-novel-5-1 | TGME49_chrIV_2285074_2285293_- | 5’ | InterGenic | N | AGATTGAGCGTGGACTATCG | 20 | 0.2 | 1.6 |
| tgo-novel-52 | TGGT1_chrX_6803298_6803464_- | 3’ | InterGenic | N | AGTGAGCTAAATAGATACAAGGAA | 24 | 1.2 | 0 |
| tgo-novel-5-2 | TGVEG_chrIV_2351262_2351481_- | 5’ | InterGenic | N | AGATTGAGCGTGGACTATCG | 20 | 0.2 | 1.6 |
| tgo-novel-53 | TGVEG_chrV_1999469_1999637_+ | 3’ | InterGenic | N | AGGGAAGTGTGTTTCAAAGAAA | 22 | 0.5 | 46.6 |
| tgo-novel-5-3 | scf_1107000998844_776_875_+ | 5’ | InterGenic | N | AGATTGAGCGTGGACTATCG | 20 | 0.2 | 1.6 |
| tgo-novel-54-1 | TGGT1_chrX_5710851_5710946_+ | 3’ | Intron | N | AACGGGACATTAGACCGAACC | 21 | 0 | 2.6 |
| tgo-novel-54-2 | TGME49_chrX_5725772_5725867_+ | 3’ | UTR | N | AACGGGACATTAGACCGAACC | 21 | 0 | 2.6 |
| tgo-novel-54-3 | TGVEG_chrX_5790797_5790892_+ | 3’ | InterGenic | N | AACGGGACATTAGACCGAACC | 21 | 0 | 2.6 |
| tgo-novel-55-1 | TGGT1_chrVIII_2699152_2699245_+ | 5’ | Intron | N | AGGAACACTTGTGAAGGAGACAT | 23 | 3 | 0 |
| tgo-novel-55-2 | TGME49_chrVIII_2699125_2699218_+ | 5’ | Intron | N | AGGAACACTTGTGAAGGAGACAT | 23 | 3 | 0 |
| tgo-novel-55-3 | TGVEG_chrVIII_2699048_2699141_+ | 5’ | Intron | N | AGGAACACTTGTGAAGGAGACAT | 23 | 3 | 0 |
| tgo-novel-56-1 | TGGT1_chrXI_2341684_2341841_- | 5’ | InterGenic | N | CGGCTTTTGGTTTGATGGAAC | 21 | 0 | 2.6 |
| tgo-novel-56-2 | TGME49_chrXI_2721857_2722014_- | 5’ | InterGenic | N | CGGCTTTTGGTTTGATGGAAC | 21 | 0 | 2.6 |
| tgo-novel-56-3 | TGVEG_chrXI_2719971_2720128_- | 5’ | InterGenic | N | CGGCTTTTGGTTTGATGGAAC | 21 | 0 | 2.6 |
| tgo-novel-57-1 | TGGT1_chrVIIa_779953_780008_+ | 5’ | InterGenic | N | ATGGTATTGGAGAAAGGAGGTT | 22 | 18.5 | 2.3 |
| tgo-novel-57-2 | TGME49_chrVIIa_777882_777937_+ | 5’ | InterGenic | N | ATGGTATTGGAGAAAGGAGGTT | 22 | 18.5 | 2.3 |
| tgo-novel-57-3 | TGVEG_chrVIIa_786478_786533_+ | 5’ | InterGenic | N | ATGGTATTGGAGAAAGGAGGTT | 22 | 18.5 | 2.3 |
| tgo-novel-6-1 | TGGT1_chrVI_505728_505826_+ | 5’ | InterGenic | N | GACGGTCTTTCTGCGAGCGTGC | 22 | 0 | 6.2 |
| tgo-novel-6-2 | TGME49_chrVI_498885_498983_+ | 5’ | InterGenic | N | GACGGTCTTTCTGCGAGCGTGC | 22 | 0 | 6.2 |
| tgo-novel-6-3 | TGVEG_chrVI_1977158_1977256_+ | 5’ | InterGenic | N | GACGGTCTTTCTGCGAGCGTGC | 22 | 0 | 6.2 |
| tgo-novel-7-1 | TGGT1_chrVIIa_4095572_4095768_+ | 5’ | UTR | N | AGGGGGCGAGAACTTGCTGAC | 21 | 0 | 1.9 |
| tgo-novel-7-2 | TGME49_chrVIIa_4076620_4076816_+ | 5’ | InterGenic | N | AGGGGGCGAGAACTTGCTGAC | 21 | 0 | 1.9 |
| tgo-novel-7-3 | TGVEG_chrVIIa_4098705_4098901_+ | 5’ | UTR | N | AGGGGGCGAGAACTTGCTGAC | 21 | 0 | 1.9 |
| tgo-novel-8-1 | TGGT1_chrVIII_737414_737586_- | 5’ | UTR | N | TAGGGAGTCTGTTGCTGGGGC | 21 | 0 | 3.2 |
| tgo-novel-8-2 | TGME49_chrVIII_736447_736619_- | 5’ | UTR | N | TAGGGAGTCTGTTGCTGGGGC | 21 | 0 | 3.2 |
| tgo-novel-8-3 | TGVEG_chrVIII_736676_736848_- | 5’ | InterGenic | N | TAGGGAGTCTGTTGCTGGGGC | 21 | 0 | 3.2 |
| tgo-novel-9-1 | TGGT1_chrVIIa_990778_990852_- | 5’ | InterGenic | N | TAGAATGATGATCGAAGT | 18 | 0 | 4.5 |
| tgo-novel-9-2 | TGME49_chrVIIa_988154_988228_- | 5’ | InterGenic | N | TAGAATGATGATCGAAGT | 18 | 0 | 4.5 |
| tgo-novel-9-3 | TGVEG_chrVIIa_998660_998734_- | 5’ | InterGenic | N | TAGAATGATGATCGAAGT | 18 | 0 | 4.5 |

^a^Y indicates that the sequences from both strands of a miRNA* species were found, while N means that only the sequence from one strand of a miRNA* was identified.

^b^The abundance value of each miRNA was normalized to “transcripts per million (TPM)”. If the value after normalization was less than 1, the normalized value was set as 1.

^c^The expression of miRNA was the most abundant sequence of the total counts of unique reads.
